# Symbiotic UCYN-A strains co-occurred with El Niño, relaxed upwelling, and varied eukaryotes over 10 years off Southern California

**DOI:** 10.1038/s43705-023-00268-y

**Published:** 2023-06-24

**Authors:** Colette Fletcher-Hoppe, Yi-Chun Yeh, Yubin Raut, J. L. Weissman, Jed A. Fuhrman

**Affiliations:** 1 Marine & Environmental Biology, Department of Biological Sciences, University of Southern California (USC), Los Angeles, CA USA; 2 Department of Global Ecology, Carnegie Institution for Science, Stanford University, Stanford, CA USA; 3 Schmid College of Science and Technology, Chapman University, Orange, CA USA

**Keywords:** Water microbiology, Biogeochemistry, Microbial ecology, Symbiosis, Biogeochemistry

## Abstract

Biological nitrogen fixation, the conversion of N_2_ gas into a bioavailable form, is vital to sustaining marine primary production. Studies have shifted beyond traditionally studied tropical diazotrophs. *Candidatus Atelocyanobacterium thalassa* (or UCYN-A) has emerged as a focal point due to its streamlined metabolism, intimate partnership with a haptophyte host, and broad distribution. Here, we explore the environmental parameters that govern UCYN-A’s presence at the San Pedro Ocean Time-series (SPOT), its host specificity, and statistically significant interactions with non-host eukaryotes from 2008-2018. 16S and 18S rRNA gene sequences were amplified by “universal primers” from monthly samples and resolved into Amplicon Sequence Variants, allowing us to observe multiple UCYN-A symbioses. UCYN-A1 relative abundances increased following the 2015-2016 El Niño event. This “open ocean ecotype” was present when coastal upwelling declined, and Ekman transport brought tropical waters into the region. Network analyses reveal all strains of UCYN-A co-occur with dinoflagellates including *Lepidodinium*, a potential predator, and parasitic *Syndiniales*. UCYN-A2 appeared to pair with multiple hosts and was not tightly coupled to its predominant host, while UCYN-A1 maintained a strong host-symbiont relationship. These biological relationships are particularly important to study in the context of climate change, which will alter UCYN-A distribution at regional and global scales.

## Introduction

Biological nitrogen fixation sustains primary production in much of the ocean. In this process, rare prokaryotes known as diazotrophs convert inert dinitrogen gas (N_2_) to ammonia (NH_3_). For many years, only a handful of well-characterized photosynthetic bacteria were thought to be capable of marine nitrogen fixation: *Trichodesmium*, *Crocospharea watsonii*, and symbionts in diatom-diazotroph associations were considered the dominant diazotrophs (e.g., [1, 2]). However, traditional paradigms of biological nitrogen fixation are continuously being challenged (e.g., [2]). Studies that used amplicon sequencing to target the gene *nifH*, which encodes a subunit of the nitrogenase enzyme that conducts nitrogen fixation, have revealed that diazotrophs are a more diverse group than previously recognized (e.g., [3–6]). The first study that applied this technique to marine organisms observed a cluster of *nifH* sequences belonging to a clade termed “UCYN-A”, for “unicellular cyanobacterial group A” [7]. This clade of organisms has been tentatively named *Candidatus Atelocyanobacterium thalassa* [8], and is now recognized as a major contributor to biological nitrogen fixation (e.g., [9, 10]).

UCYN-A is an aberrant cyanobacterium, lacking Photosystem II and key components of cellular pathways, such as the Krebs cycle [11]. Its metabolism is streamlined because it lives in symbiosis with a photosynthetic haptophyte host, exchanging fixed nitrogen for carbon compounds [8, 12]. Four clades of UCYN-A are currently recognized based on their *nifH* sequences, although more may exist [13]. UCYN-A1, the most extensively studied type of UCYN-A, associates with a coccolith-forming member of the genus *Braarudosphaera* [8], and is found primarily in open-ocean regions [14, 15]. UCYN-A1 is <1 μm in diameter while host cells have a diameter of 1–3 μm and can house 1–2 symbionts each [13, 16]. UCYN-A2, a coastal ecotype [14, 15], associates with *Braarudosphaera bigelowii*, also coccolith-forming [12, 17], and is larger than 1μm, while host cells are 4–10 μm in diameter (e.g., [18]). The UCYN-A2 host likely has more symbiont per cell: although microscopy suggests UCYN-A2 has one symbiont per host cell [19], DNA sequencing shows the UCYN-A2 host can house 4-10 symbionts per cell [14, 16].

UCYN-A has a broad, global distribution [9, 13], including nitrogen-rich regions such as coastal and equatorial upwelling systems (e.g., [2, 10, 20, 21]). UCYN-A1 and UCYN-A2 have been found previously in the Southern California Current System and Monterey Bay (primarily UCYN-A2) [22, 23]. Notably, UCYN-A has been observed at our study site, the San Pedro Ocean Time-series (SPOT), as deep as 890 m [24], and at a nearby daily time series off the coast of Catalina Island [25]. Furthermore, UCYN-A was found to comprise up to 95% of the diazotroph population sampled from San Diego to Sebastian Vizcaino Bay (Baja, CA) and within the period of our 10-year timeseries (2008–2018) [22].

Nitrogen fixation by many species has been observed in coastal ecosystems in surprisingly high rates, often despite high concentrations of available nitrogen, and high relative abundances of eukaryotes which may outcompete them. Nitrogen fixation off the New Jersey Shore may support up to 100% of primary production in this ecosystem, with some of the highest reported UCYN-A abundances [26]. Coastal nitrogen fixation in Southern California has been measured at lower rates (e.g., [22, 23]). Few studies have attempted to link diazotrophs to potential eukaryotic predators, and these reports have focused on diazotrophs confined to the tropics [27, 28].

In addition, many questions remain about the UCYN-A symbiosis, including the specificity of host-symbiont partnerships (e.g., UCYN-A has been reported without a host [18]). The question of UCYN-A host specificity is especially important because ocean acidification may degrade the calcareous shells of the established hosts during the coccolith-bearing phases of their life cycles [23, 29]. This in turn could alter the global distribution of the symbiont and its global contributions to biological nitrogen fixation.

DNA sequencing within  a long timeseries project, as we report here, is a particularly valuable method for studying microbial interactions and changes therein. In our time series, genes encoding the small subunit of ribosomal RNA (i.e. 16S rRNA genes (“16S”) for prokaryotes and 18S rRNA genes (“18S”) for eukaryotes) of the entire microbial community were processed into Amplicon Sequence Variants (ASVs) using “universal” primers that capture all three domains of life [30–32]. Because we did not design the study with a particular set of organisms in mind, we can examine these data for co-occurrences between any microbes, which may suggest biological interactions. Each set of DNA sequences from a community provides a snapshot into its structure at the time of sampling. Time series projects, in which communities are sampled on a regular basis over years, allow researchers to assemble a “movie” of what entire microbial communities are actually doing over time [33], including looking at events that occurred only rarely. In this study, we sought to characterize the abiotic niche of UCYN-A, its potential predators, and its host specificity, using 16S & 18S ASVs over a decade+-long time series in coastal, temperate waters.

## Materials and methods

### Data collection

**Fig. 1:**
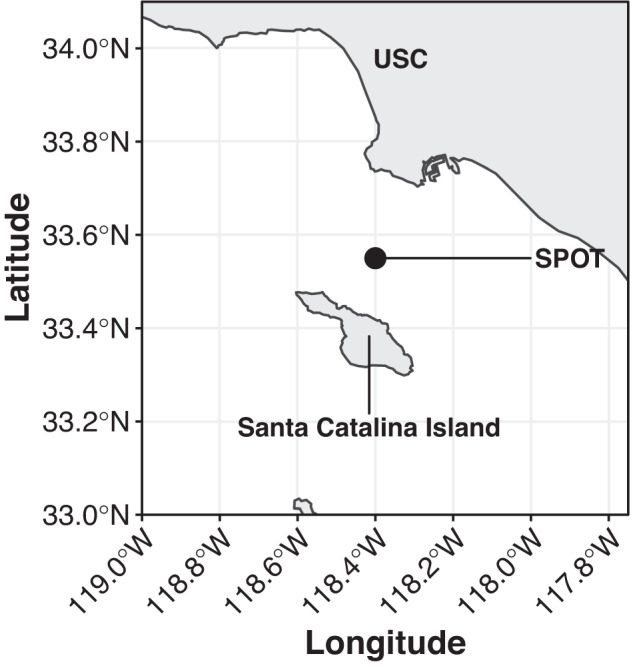
Sampling site location for the San Pedro Ocean Time-series (SPOT). SPOT is ~16 km from the Port of Los Angeles (33.55°N, 118.4°W). USC, University of Southern California. Sample collection is described in detail in Yeh et al. [33].

Seawater was collected monthly from 5m (surface) and deep chlorophyll maxima (DCM) from 2000 to 2018 at the San Pedro Ocean Time-series (33.55°N, 118.4°W; Fig. 1) (although statistical analyses represent data from 2008 to 2018). Samples were collected and processed into 16S and 18S ASVs, which differ by as little as one base pair, using a wrapper of the software Quantitative Insights Into Microbial Ecology v2 (QIIME2) ([30, 34], also see supplemental methods for more details). Because we only wished to examine interactions between whole, single-celled organisms, ASVs corresponding to chloroplasts and multicellular metazoans were removed from the analyses reported here.

To investigate host specificity, relative abundances of UCYN-A ASVs and their known hosts were normalized to a common denominator and compared (see supplemental methods).

Upwelling intensity at 33°N, measured by the Biologically Efficient Upwelling Transport Index (BEUTI) and Coastal Upwelling Transport Index (CUTI) [35], were downloaded from the National Ocean and Atmospheric Administration’s (NOAA’s) Pacific Fisheries Environmental Laboratory (https://oceanview.pfeg.noaa.gov/products/upwelling/cutibeuti). Components of the Bakun Index for upwelling at the nearest available point (33.5°N, -118.5°W) were also downloaded from NOAA (https://coastwatch.pfeg.noaa.gov/erddap/griddap/erdlasFnWPr.html). Multivariate ENSO Index (MEI) data, indicative of El Niño (positive MEI)/La Niña (negative MEI), were obtained from the NOAA Physical Sciences Laboratory (https://psl.noaa.gov/enso/). As recommended, each bimonthly sliding window was used to represent the latter of the two months. Bacterial production was measured by incorporation of tritiated leucine (e.g., [36]). Inorganic nitrogen ([NO_2_^−^ + NO_3_^−^]) and phosphate ([PO_4_^3−^]) concentrations at the time of sampling were measured via a Lachat spectrophotometer QuickChem 8500 Series 2 at the Marine Science Institute at the University of California, Santa Barbara (concentration range 0.2–300 µM for nitrogen, 0.1–200 µM for phosphate).

### Phylogenetic tree

The QIIME2 classifier identified six 16S ASVs as UCYN-A with >99.9% confidence and identified seven 18S ASVs as *Braarudospharea* with >93% confidence. The sequences for these ASVs, along with published 16S sequences of UCYN-A and 18S sequences of *Braarudospharea*, were assembled into phylogenetic trees for host and symbiont. Trees included all UCYN-A and *Braarudospharea* sequences publicly available as of January 2022. Sequences were aligned via maaft with default settings [37]. The alignment was trimmed via trimAl, also with default settings [38]. Trees were constructed via Randomized Accelerated Maximum Likelihood with rapid bootstrap analysis and 100 bootstraps [39], and visualized via the interactive Tree of Life (iTOL [40]).

### Relationship with abiotic factors

Logistic regression was used to evaluate effects of each environmental parameter on UCYN-A symbiont and host presence at SPOT. This process was repeated for a *Lepidodinium* ASV. The mean and standard error of each parameter were plotted on days that each organism was present or absent (defined as >0.01% of the 16S community) via ggplot 2 [41]. Statistical significance of these differences was corrected for multiple testing via Benjamini-Hochberg correction.

For all other analyses, relative abundance data were prepared as follows. Five samples from the 5m depth and two samples from the DCM were excluded because they contained too few sequences to capture the diversity of 18S ASVs at SPOT (Fig. S1). Abundance data from missing dates were linearly interpolated via na.approx() from the R package zoo [42]. To avoid common problems with compositional data [43, 44], relative abundances were centered log-ratio (CLR) transformed with the mclr() function from the SPRING package [45].

Spearman’s rank-order correlation was used to evaluate the monotonic relationship between environmental variables and CLR-transformed host/symbiont relative abundances. Spearman’s correlation was performed using the rcor() function with type = “spearman” from the Hmisc package in R.

Additional analyses are described in Supplementary Methods.

### Network analyses

Co-occurrence networks were generated on interpolated, CLR-transformed data with extended local similarity analysis (eLSA; [46, 47]). Networks constructed using non-CLR transformed data missed several correlations detectable from transformed data (Fig. S2). This study reports on associations between UCYN-A and 18S taxa at 5m from March 2008 to July 2018. CLR-transformed UCYN-A1 relative abundances from the smaller size fraction were included on all dates. On several dates that UCYN-A1 and UCYN-A2 relative abundances peaked in the larger size fraction prokaryotic community, the 100 most abundant eukaryotic taxa were selected for inclusion in network analyses. eLSAs were run with 1000 permutations and default normalization off. *Q* values were calculated from P-values using the qvalues() package (e.g., [48]). Correlations at the 5m depth that were highly statistically significant via both Pearson’s correlation and Spearman’s correlation (all *P* values < 0.005 and all *Q* values < 0.01) were visualized using Cytoscape v3.5 [49]. Relative abundances of the 18S taxa included in these networks were visualized via Krona plots.

## Results

### Phylogenetic tree

Six 16S ASVs were classified as UCYN-A with >99.99% confidence according to QIIME2.

(QIIME2 classifies ASVs based on SILVA 132 and assigns each a unique identifier or hash (#); ASV numbers reflect the alphabetical order of the hashes QIIME2 generated).

**Fig. 2:**
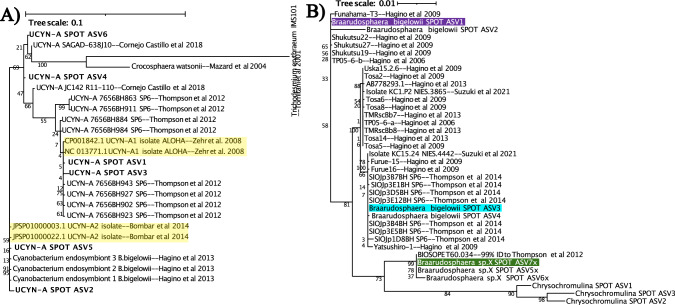
Established 16S and 18S sequences of UCYN-A and host ASVs match SPOT ASVs. UCYN-A 16S sequences (**A**) and *Braarudosphearea* 18S sequences (**B**) from SPOT are phylogenetically identical to the reference sequences for UCYN-A1, UCYN-A2, and hosts shown to associate with these symbionts. Bolded names indicate UCYN-A sequences from SPOT; yellow highlighted names indicate 16S sequences from the published genomes of symbionts. *Braarudospharea* ASVs that associate with UCYN-A are bolded and highlighted in green, purple, and blue. Trees were generated via RAxML with 100 bootstraps; numbers indicate bootstrap values.

One ASV (#3d852410f44d21c92c9c55fbbb25187e) matched the published genome of the UCYN-A1 sublineage perfectly (100% BLAST identity, also see Fig. 2A, Table S1; [50]), and will be referred to as UCYN-A1. Another 16S ASV (#af1bb1f9fb1c3f3d18571e711df407bb) matched the published genome of the UCYN-A2 sublineage (100% BLAST identity, also see Fig. 2A, Table S1; [14]). In addition, this ASV never appeared in the smaller size fraction of filters (0.22–1 μm), which is consistent with the reported larger diameter of UCYN-A2 (>1 μm) [51]. This ASV will be referred to as UCYN-A2.

QIIME2 classified seven of the 18S ASVs found at SPOT as *Braarudosphaera*, the genus that contains previously established hosts of UCYN-A symbionts, with >93% confidence. Four of these seven ASVs were classified as *B. bigelowii*, the putative host of UCYN-A2. *B. bigelowii* ASV1, ASV3, and ASV4 (#04926e2fd1b8706b4866c02650f702dd, #70a5283da28db501a349c5beb22881e7, #8c144683114fbb1ad2e9425f7dcd1b02, respectively) closely matched strains of *B. bigelowii* shown to associate with UCYN-A2 (Fig. 2B, Table S1; [12]). The other three *Braarudospharea* ASVs did not match a formally named species, and are referred to as 5x, 6x, and 7x. Of these, *Braarudosopharea* ASV7x matched isolate BIOSOPE T60 (100% BLAST identity, also see Fig. 2B, Table S1), the established host of the published UCYN-A1 genome [8].

### Relationship with abiotic factors

**Fig. 3:**
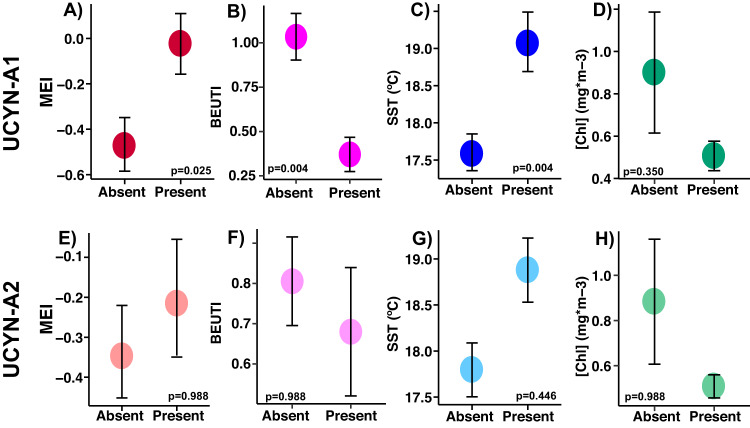
UCYN-A1 is present at SPOT under El Niño conditions, with relaxed upwelling, higher temperatures, and lower biomass. On dates UCYN-A1 is present at SPOT, MEI is higher, upwelling is weaker, surface waters are warmer, and biomass is lower (**A**–**D**); these patterns are similar but weaker for UCYN-A2 (**E**–**H**) (note larger *p* values and error bars). Differences in the mean of each value when ASVs were present or absent were evaluated via logistic regression. *P* values reported were corrected for multiple testing via Benjamini-Hochberg correction. Error bars represent standard error. Higher Multivariate ENSO Index (MEI) indicates El Niño conditions (more stratified); higher Biologically Effective Upwelling Transport Index (BEUTI) indicates stronger upwelling. Additional variables are shown in Supplementary Table 1, including statistically significant differences in upwelling (measured by Coastal Upwelling Transport Index (CUTI), bacterial production, and currents on days UCYN-A were present vs. absent.

**Fig. 4:**
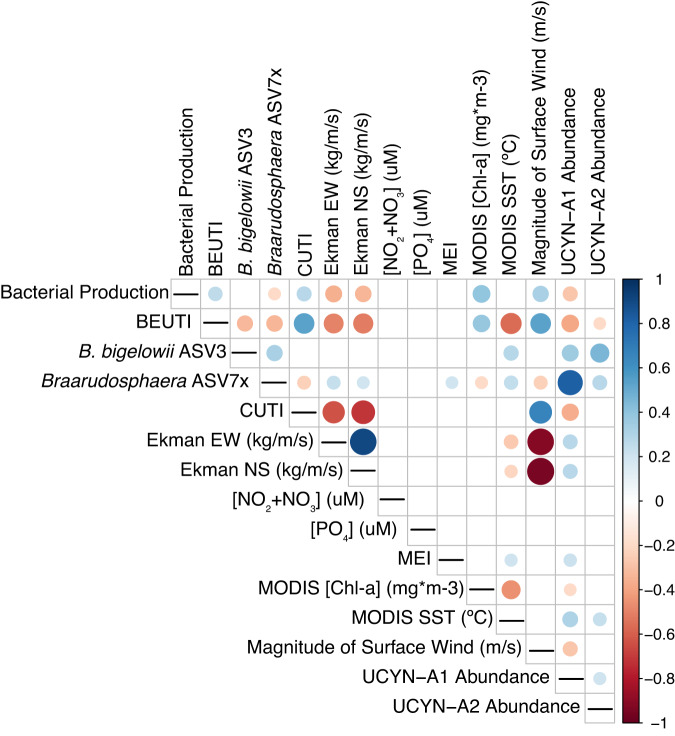
UCYN-A1 relative abundances correlate positively with host organisms, UCYN-A2 host/symbionts, MEI, and SST, and negatively with upwelling indices (BEUTI and CUTI) and indirect indicators of upwelling, including bacterial production, East-West Ekman transport, and chlorophyll concentrations. UCYN-A symbiont/host ASVs were CLR-transformed and correlated with environmental parameters via Spearman’s rank-order correlation. Dot size indicates the strength of correlation, while dot color represents positive or negative associations. Only statistically significant correlations (*p* < 0.05) are shown via dots; correlations between variable pairs that are insignificant or that do not correlate are represented by blank squares. BEUTI Biologically Effective Upwelling Transport Index, CUTI coastal upwelling transport index, EW East-West, NS North-South, MEI Multivariate ENSO Index.

Regional coastal upwelling, measured by BEUTI, was significantly weaker on dates that UCYN-A1 was present (Fig. 3, Table S2). In addition, chlorophyll-a concentrations and bacterial production were lower when UCYN-A1 was present, while sea surface temperature (SST) and MEI were higher (Fig. 3). Ekman transport moved surface water north and east during months UCYN-A1 was present at SPOT (Table S2). UCYN-A1 relative abundances correlated positively with MEI and SST, and negatively with bacterial production, upwelling, and chlorophyll-a (Fig. 4). Higher than average relative abundances of UCYN-A1 coincided with low upwelling indices, positive MEI, and SST > 19 °C (Fig. S3). On dates UCYN-A2 was present, the BEUTI index was slightly lower (Fig. 3). Relative abundances of UCYN-A2 showed a weak positive correlation with SST and a weak negative correlation with BEUTI (Fig. 4), but no significant correlation to chlorophyll concentrations, bacterial production, SST, and MEI (Table S2). Neither nitrogen nor phosphorus concentrations differed significantly on dates that UCYN-A ASVs were present vs. absent (Table S2).

### Co-occurrence with non-host 18S taxa

**Fig. 5:**
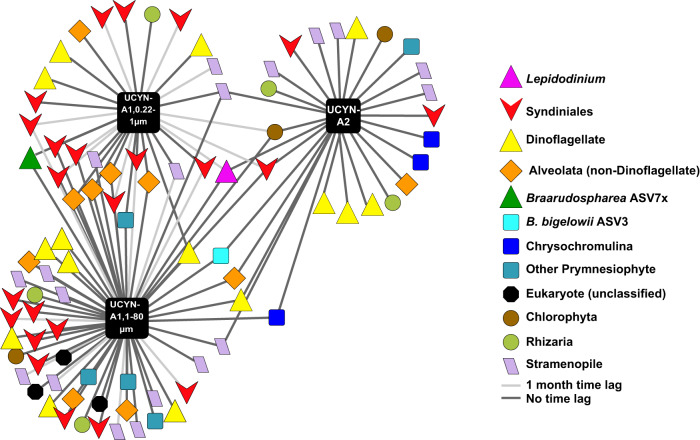
UCYN-A co-occurs with a variety of 18 S taxa at the SPOT surface, notably including *Lepidodinium*, hypothesized to be a predator (pink triangle, center). Networks were generated via eLSA and visualized in Cytoscape 3.5. Each node represents one ASV; only ASVs that co-occurred with *P* < 0.005 and *Q* < 0.01 by both Pearson’s and Spearman’s correlation are shown using easily recognizable names. QIIME-generated hashes of each node are presented in Table S3; 16S and 18S sequences of each node, including UCYN-A symbionts, are publicly available (see Data Availability Statement).

With high statistical significance (*P* < 0.005 and *Q* < 0.01 by Local Similarity, Pearson’s and Spearman’s correlation), UCYN-A co-occurred with numerous 18S ASVs, such as prymnesiophytes and Dinoflagellates, notably including parasitic Syndiniales and an ASV from the genus *Lepidodinium*. UCYN-A1 and UCYN-A2 shared several of these taxa in common (Figs. 5, S4, Table S3). The *Lepidodinium* ASV was present at SPOT under environmental conditions similar to those found when UCYN-A ASVs were present (Table S2).

### UCYN-A host-symbiont co-occurrences

**Fig. 6:**
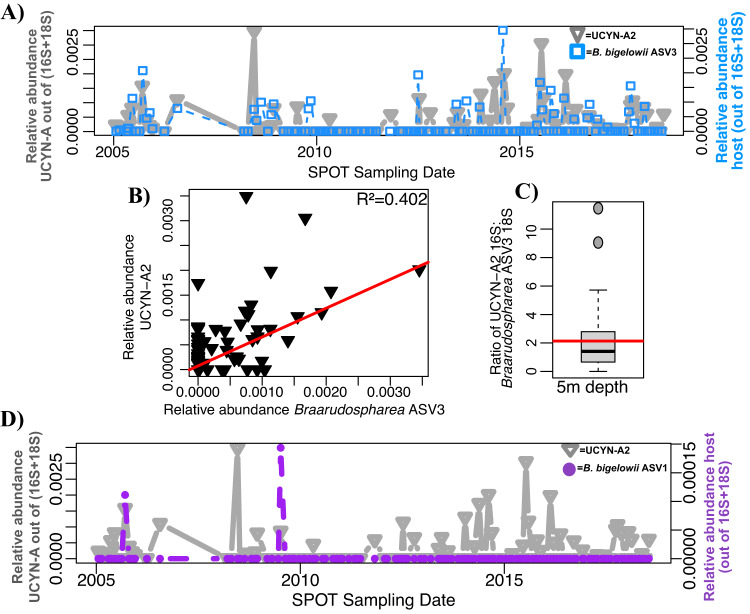
UCYN-A2 is less tightly coupled to its prymnesiophyte host than the UCYN-A1 symbiosis. **A** UCYN-A2 often, but not always, co-occurs with its established host at 5 m depth across the SPOT time series. **B** UCYN-A2 relative abundance correlates with relative abundance of its most common host more weakly than the UCYN-A1 symbiosis. **C** The ratio of 16S: 18S genes of these organisms is lower than expected. Boxplot values indicate the median and interquartile range values of this ratio; the red line indicates the average (2.139). 29 sampling dates, on which host and symbiont are present, are included. **D** UCYN-A2 occasionally co-occurs with *Braarudospharea* ASV1. The relative abundances of 16S and 18S ASVs were normalized as described in Supplementary Methods to allow for direct comparison.

UCYN-A2 co-occurred with *B. bigelowii* ASV3 on ~60% of the dates it was present at the SPOT surface (Figs. 6A–C, S5, S6). On average, the ratio of UCYN-A2 16S: *B. bigelowii* ASV3 18S was about 2:1 at the SPOT surface (Fig. 6C). Another ASV of *B. bigelowii*, ASV1, peaked in relative abundance on dates where UCYN-A2 was present, but *B. bigelowii* ASV3 was absent (Fig. 6D). At the DCM, *Braarudospharea* ASV3 was present on ~60% of the dates UCYN-A2 was present, but the ratio of their 16 S: 18 S genes of these organisms was about 1:1 on average (Fig. S6).

**Fig. 7:**
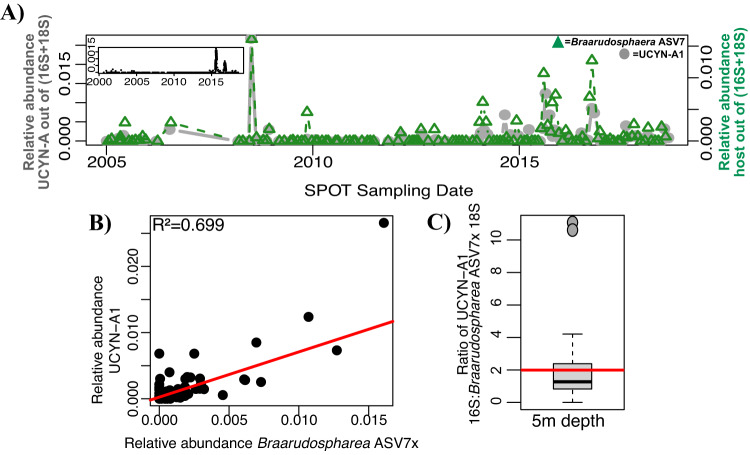
UCYN-A1 and its prymnesiophyte host are tightly coupled throughout the SPOT dataset. **A** UCYN-A1 consistently co-occurs with its putative host at 5 m depth across the SPOT time series. Panel inset indicates the relative abundance of UCYN-A1 in the smaller size fraction. **B** UCYN-A1 relative abundance strongly correlates with its putative host relative abundance. **C** The ratio of 16S: 18S genes of these organisms is, on average, as expected. Boxplot values indicate the median and interquartile range (IQR) values of this ratio; the red line indicates the average (1.991). One outlier (ratio > 50) was excluded. 43 sampling dates are included. As with UCYN-A2, the relative abundances of 16S and 18S ASVs were normalized to allow for direct comparison.

The relative abundance of UCYN-A1 closely mirrored that of *Braarudosphaera* ASV7x, the closest match for the known host of UCYN-A1 (BIOSOPE T60; Fig. 2B), at the SPOT surface (Figs. 7A–C, S5, S7). The 16S gene of UCYN-A1 and 18S gene of *Braarudosphaera* ASV7x consistently co-occurred in nearly a 2:1 ratio at 5 m depth (Fig. 7C). In addition, UCYN-A1 co-occurred with *Braarudosphaera* ASV7x in eLSA networks with high statistical significance at the SPOT surface (Fig. 5). These organisms mirror each other less strongly at the DCM, where their ratio of 16 S: 18 S genes is also ~2:1 (Fig. S7).

### Spatial-temporal distributions of UCYN-A ASVs

UCYN-A ASVs were primarily found in the larger size fraction in the euphotic zone; one was also detected once at 890m at about 0.5% of the 16S community (Figs. S8, S9). UCYN-A1 was found on 44% of sampling dates, while UCYN-A2 was present at the SPOT surface on 38.4% of all sampling dates (Figs. S3, 6A, 7A). Relative abundances of all *Braarudosphaera* ASVs were highest in the euphotic zone, and they appeared at depths greater than the DCM even more rarely than UCYN-A.

## Discussion

### Relationship with abiotic factors

Currents flowing from the tropical Pacific to the north and east likely brought the UCYN-A1 symbiosis, the “open ocean” ecotype, into our study system. These currents are particularly strong during El Niño conditions and less so during regional upwelling. Positive Multivariate ENSO Index (MEI) values indicate El Niño conditions, in which warm, tropical waters flow west to east across the equator, altering climate patterns throughout the Pacific Ocean and weakening upwelling (e.g., [52, 53]). UCYN-A1 presence and relative abundance at our study site are positively correlated with higher MEI, i.e., El Niño conditions (Fig. 3, Table S2, Figs. 4,  S4). Our data show a sudden increase in the relative abundances of UCYN-A1 and host following the 2015-2016 El Niño event, which were sustained in the subsequent years (Fig. 7A). Similarly, other time-series data show offshore ASVs were advected into the Southern California Bight after the same El Niño event [54], including warm ecotypes of *Prochlorococcus* at our study site [34]. Prior to this event, e.g., in 2010-2011, the UCYN-A1 symbiosis was infrequently detected at our sampling site and was similarly absent from other locations within the Southern California Current during overlapping periods of sampling [20, 22].

In addition, multiple lines of evidence suggest that UCYN-A1 presence and relative abundance are negatively influenced by seasonal upwelling. In this annual, coastal event, Ekman transport displaces surface waters west and south from SPOT, and nutrient-rich waters rise from the deep to replace them, stimulating increased bacterial chlorophyll concentrations and productivity (e.g., [35, 55]). Upwelling is here measured by the BEUTI and CUTI indices [35], with lower BEUTI and CUTI values indicating weaker upwelling. Lower BEUTI and CUTI are correlated with UCYN-A1 presence (Fig. 3, Table S2), while sparse binomial regression indicated that the CUTI index negatively affected UCYN-A1 presence at SPOT (see Supplemental Information). Indirect indicators of upwelling, including Ekman transport to the south and west, lower SST, higher bacterial production rates and [Chl-a], were also negatively correlated with UCYN-A1 presence and abundance (Fig. 3, Table S2, Figs. 4, S4). In Monterey Bay, north of our study site, both UCYN-A1 and UCYN-A2 (the dominant sublineage at this site), also regularly appeared in August-October, after regional upwelling had ceased [23]. Previous studies have also related UCYN-A1 abundances to temperature: although UCYN-A distribution may not be directly influenced by temperature (e.g., [9, 18]), warmer waters are favorable to increased UCYN-A1 relative abundance [22, 23]. In tandem, these observations indicate that the “open ocean” ecotype of the UCYN-A symbiosis is advected into our study system by warm, tropical waters during El Niño and periods of relaxed upwelling, which foster lower biomass and coincide with higher temperatures, providing ideal conditions for the open-ocean ecotype.

UCYN-A1 relative abundances and nitrogen fixation rates were observed to increase days after upwelling in nearshore samples from the Scripps Pier and the Alaskan Beaufort shelf [22, 56]. The daily sampling resolution of these cruises or their proximity to shore may explain discrepancies with our study. Upwelling may stimulate UCYN-A seed populations at a daily timescale, perhaps due to community production of a micronutrient not measured here that is essential to the UCYN-A1 symbiosis [22, 57, 58]. SPOT data, which is collected monthly, may not capture these short-term dynamics. Instead, we observe that upwelling hinders UCYN-A proliferation. Notably, both previous studies collected samples solely in 2017, soon after the 2015-2016 El Niño event. This could have brought UCYN-A seed populations to the Scripps Pier study, which was concurrent with an influx of warm, low-density waters from the tropics. Our study shows that over longer time scales, ocean currents including this El Niño event and relaxed upwelling created warmer, low biomass conditions which were conducive to UCYN-A1.

Differences between UCYN-A1 and UCYN-A2 symbioses regarding abiotic niches support the hypothesis partitioning them into coastal and open-ocean ecotypes (e.g., [14]). UCYN-A2 presence was not strongly affected by seasonal upwelling or El Niño (Fig. 3), and its relative abundance was only correlated with two abiotic factors: BEUTI index (negative) and SST (positive) (Fig. 3, Table S2, Fig. S4).

Given the paradigm presented here, climate change will likely have a strong but mixed effect on UCYN-A1 at SPOT. Intense El Niño events are expected to become more frequent with climate change [57], advecting warm, tropical waters, along with UCYN-A1, into Southern California more often. Simultaneously, upwelling events along the California Current System are expected to intensify, as warming on land increases air pressure differences between land and sea, strengthening coastal winds and upwelling [55, 59, 60]. Notably, the degree to which upwelling may intensify is unclear [60], partially due to mitigative effects of El Niño events, which weaken upwelling [53]. Although upwelling deters UCYN-A1 proliferation at SPOT, the symbiosis was able to recover from upwelling events following the 2015-2016 El Niño event (Fig. 7). Future populations of UCYN-A1 may adhere to this trend, such that the symbiosis reaches greater abundances following future El Niño events. Because UCYN-A symbioses comprise up to 95% of the nitrogen fixing population in Southern California, this could substantially increase inputs of fixed nitrogen [22]. Notably, inputs of fixed nitrogen are small in this region [22], but this may be due to eukaryotic grazing that rapidly consumes diazotrophs as they fix nitrogen [10].

### Co-occurrence with non-host 18 S taxa

Based on co-occurrence data with high statistical significance (Figs. 5, S4, Table S3), we hypothesize a predator-prey relationship between the UCYN-A symbiosis and *Lepidodinium*, which in turn may be parasitized by Syndiniales (Fig. S4). The dinoflagellate genus *Lepidodinium* has been shown to prey on the tropical diazotroph *Crocosphearea watsonii* through Lotka-Volterra modeling of environmental data [28], and doubles its grazing rates at night, when *C. watsonii* organism fixes nitrogen [27]. Furthermore, *Lepidodinium* has been hypothesized as a grazer of UCYN-A symbioses, as the diameter of UCYN-A symbioses (1-3 µm for UCYN-A1 symbioses and 4-10 µm for UCYN-A2 symbioses) are similar to that of *C. watsonii* (3.9 µm for *C. watsonii* [27]). Its appearance in our dataset supports the hypothesis that this genus could prey on UCYN-A and other nitrogen fixers. The order Syndiniales is primarily known to parasitize dinoflagellates (e.g., [61–64]. Syndiniales ASVs most likely co-occur with UCYN-A indirectly, due to the presence of their dinoflagellate hosts (see Fig. S4). However, it is possible that Syndiniales are parasitizing *Braarudospharea* directly, as some Syndiniales are able to parasitize non-dinoflagellate hosts (e.g., [65]). Our co-occurrence data and others show statistically significant co-occurrence links between haptophytes and Syndiniales (Figs. 5,  S4, Table S3 [65]).

Many of the associations discussed here have been reported previously. The paper that announced *Braarudosphaera* as the putative host of UCYN-A1 used a UCYN-A *nifH* probe and FACS (Fluorescent-Activated Cell Sorting) to show that UCYN-A physically associates with many families, including Dinophyceae, the family that contains Dinoflagellates [8]. Using 18S primers, Krupke et al. found that UCYN-A co-occurs with Rhizaria, Dinoflagellates, including *Lepidodinium* and Syndiniales, and prymnesiophytes including *Chrysochromulina*, which is taxonomically very close to (and in some cases may overlap with) *Braarudospharea* [66]. One recent study proposed that the prymnesiophyte called *Chrysochromulina parkaea* is a strain of *B. bigelowii* (99.87% identical 18S sequence to a *Braarudosphaera* sequence) and is also able to associate with UCYN-A [19].

It should be noted that co-occurrence of taxa does not imply a biological association [67]. It is possible that UCYN-A and *Braarudosphaera* do not interact with any of the taxa reported here, but happen to co-occur due to a mutually favorable environmental niche. Notably, the *Lepidodinium* ASV is present at SPOT under similar conditions as UCYN-A1 and UCYN-A2 (Table S2). However, few of these parameters differed significantly on dates *Lepidodinium* was present vs. absent, and it is unclear if they influence the organism’s relative abundance. Additional studies should be performed to elucidate true interactions. For example, CARD FISH with labeled probes for *Lepidodinium* and for UCYN-A could reveal physical evidence of predation. To tease apart other potential interactions, multi-stressor grow-out experiments could be conducted, in which temperature and availability of key nutrients are each varied along a gradient (e.g., [68]).

### UCYN-A host-symbiont co-occurrences

The relationship between UCYN-A2 and host appears inconsistent from ASV co-occurrences. UCYN-A2 was present at the SPOT surface more frequently and in higher relative abundances than its presumed host, *B. bigelowii* ASV3 (38.4% vs. 28% of sampling dates, Figs. 6, S5). The expected ratio of symbiont 16S: host 18S rRNA genes is unclear. Microscopy images show one symbiont per cell [19]. Based on DNA sequencing data, the UCYN-A2 host is thought to have 30-40 copies of the 18S rRNA gene [14], and 7-10 symbionts with two rRNA genes each [16], such that the ratio of UCYN-A 16S rRNA genes: 18S *Braarudosphaera* rRNA genes should be about 0.66 at most. We do not expect a constant ratio or a linear relationship between host 18S and symbiont 16S genes, due to polyploidy in the host or symbiont, differences in copy number of host rRNA genes, or different stages of cell division for symbiont or host. However, the average ratio of sequences was higher than expected (average = 2.729), and their genes showed poor correlation (Fig. 6B-C).

We also observe that the same UCYN-A2 symbiont co-occurs with another potential host, *B. bigelowii* ASV1. On two days in particular, UCYN-A2 peaked in relative abundance when the abundance of its presumed host, *B. bigelowii* ASV3, was lower than expected. On both of these days, and on no other days, *B. bigelowii* ASV1 also peaked in relative abundance (Fig. 6C). Both *B. bigelowii* ASV1 and ASV3 are closely related to genotypes of *Braarudosphaera* found to associate with UCYN-A2 in the coastal waters of Japan. Hagino et al. used full length 18S sequencing, CARD FISH, and electron microscopy to observe that multiple cryptic species of *B. bigelowii* from distinct habitats amid the Japanese islands possessed UCYN-A symbionts [12, 17]. Notably, *B. bigelowii* ASV1 and ASV3 cluster with different pseudo-species: *B. bigelowii* ASV1 clusters with Intermediate form 1A, Genotype I, while *B. bigelowii* ASV3 clusters with Large form, Genotype IV [17] (Fig. 2B). This shows that the 18S gene fragments used here can differentiate between cryptic species, and suggests that the UCYN-A2 ASV we observed may have more than one host. It may be that the UCYN-A2-*B. bigelowii* ASV1 partnership is the dominant symbiosis at a different location–consistent with the observation that different *B. bigelowii* cryptic species dominate different local environments in Japan [12]–and was brought to SPOT certain dates. It is also possible that these two hosts have different UCYN-A symbionts that are identical over the portion of 16S that we sequenced: full length sequencing of the symbiont 16S rRNA gene may differentiate two closely related symbionts.

Whether or not the SPOT UCYN-A2 symbiont has two partners, UCYN-A2 and its established host are present at SPOT under different environmental conditions. The UCYN-A2 symbiont was present on days with higher concentrations of phosphate and increased upwelling (currents flowing to the south and west) compared to its host, although these differences are not statistically significant (Table S2). Culture-based studies also support the idea of weak coupling between UCYN-A2 symbiont and host: the UCYN-A2 host appears able to dissociate from its N_2_-fixing symbiont, and transcriptomics suggest it may instead rely on predation as a nitrogen source [19].

In contrast, UCYN-A1 presence more closely paralleled that of its established *Braarudosphaera* host. On >75% of days UCYN-A1 or *Braarudosphaera* ASV7x were present at SPOT, the two organisms were present in an average ratio of 2.176 (Figs. 7, S5), close to the expected 2:1 ratio of UCYN-A1 16S: *Braarudosphaera* 18S rRNA genes (UCYN-A1 has two 16S rRNA genes and its host is thought to have one copy of the 18S rRNA gene based on its small biomass [14, 16]). Each organism peaked in relative abundance only on days that its partner was also present. Conversely, on dates that one half of the pair was present but the other absent, the organism was only present in low abundances (Fig. 7). These two organisms were present under similar environmental conditions (Table S2), and co-occurred with high levels of statistical significance (Fig. 5). Many studies have shown a tight metabolic partnership between UCYN-A1 and its haptophyte host (e.g., [51, 69]), evidence that this host-symbiont pair are unable to survive without each other. This study shows that UCYN-A1 and its symbiont are seldom found apart, adding to this existing evidence.

Another ASV from the UCYN-A1 sublineage, SPOT UCYN-A ASV6, was also present at the SPOT surface (Fig. S3), but did not co-occur with any *Braarudosphaera* ASVs at any level of statistical significance (Figs. S7, S8, S10). Other studies have reported free-living UCYN-A1 symbionts in open-ocean regions but attributed this phenomenon to host-symbiont dissociation during sample collection [18]. However, these studies used CARD-FISH and clustered 16S sequences into operational taxonomic units (OTUs) rather than ASVs, methods that miss the high-resolution differences between 16S ASVs from the same clade of organisms. This further illustrates the utility of high-resolution 16S ASVs (e.g., [25]).

### Spatial-temporal distributions of UCYN-A ASVs

As expected, UCYN-A ASVs were primarily found at 5m and the DCM throughout the time series (Figs. S3, S9), consistent with reports that its host is photosynthetic [8]. However, UCYN-A1 was found as deep as 890 m depth (Fig. S9) at around the same time prior studies noted the UCYN-A *nifH* gene at the bottom of the San Pedro channel [25]. This strengthens existing evidence of the host-symbiont organisms’ capacity to export calcareous carbonate and fixed nitrogen from the euphotic zone [8], although this study does not address whether nitrogen fixation occurs at these depths. *Braarudosphaera* ASVs were found almost exclusively in the euphotic zone; the limited depth range of this genus is unique amongst Prymnesiophytes [9, 18].

Both the coastal and open ocean ecotypes of UCYN-A were present at SPOT, reflective of the fact that SPOT is an open ocean sampling site but is close enough to shore to be influenced by coastal dynamics, [70]. UCYN-A1 appeared in 44% of all large size fraction samples collected from 5m depth at SPOT (1–80 μm), and often in relative abundances as high as 2.5% of the whole community (Figs. S3, 7). On several occasions, UCYN-A1 was found in very low relative abundances in the smaller size class of organisms (0.22–1 μm) (Fig. 7A, inset). The UCYN-A1 symbiont is known to dissociate from its host, most likely due to the gentle pressures of sample filtration, which explains why it is found in multiple size fractions [11].

UCYN-A2 appeared in almost as many SPOT surface samples as UCYN-A1 (38.4%), but in lower relative abundances, at most 0.3% of the community (Figs. S3, 6). This ASV was never found in the smaller size fraction, which further indicates that the ASV is from UCYN-A2: UCYN-A2 organisms are reported to have a diameter >1 μm [51] and should consistently remain in the larger size fraction (1–80 μm). The UCYN-A2 symbiosis was outnumbered by its Clade 1 counterpart at our study location: both were present on ~40% of sampling dates (Figs. S3, 6A, 7A) and co-occurred with one another (Figs. 4,  S6), but UCYN-A1 was present at almost an order of magnitude higher relative abundance than UCYN-A2 (at most 2.5% of the community vs. 0.3%; Figs. 6A, 7A). Similar patterns were seen in the same current system, but south of our study site: UCYN-A2 symbioses were present at consistently low abundances, while UCYN-A1 symbioses had well defined seasonal patterns [22]. This may be because the “coastal ecotype” is accustomed to living in environments with more biomass than is typically present at SPOT, and the study location is more comparable to environments preferred by the “open ocean ecotype.”

## Conclusions

This paper reports trends in the spatio-temporal dynamics of the symbiotic diazotroph UCYN-A, its haptophyte hosts, and other associated taxa over ten years off the California Coast. We present important differences between two highly studied clades of UCYN-A with regards to host-symbiont relationships, co-occurrences with potential predators, and abiotic parameters. Studying these changes is particularly important as climate change continues to alter the distributions of UCYN-A, its hosts, and other associated 18S taxa [2].

## Supplementary information

Supplementary Information

## Data Availability

Forward and reverse reads from each sample in the San Pedro Ocean Time-series (SPOT) are available at EMBL under accession number PRJEB48162 and PRJEB35673, as described by Yeh and Fuhrman [34]. Scripts necessary to reproduce the analysis are available at https://github.com/jcmcnch/eASV-pipeline-for-515Y-926R [31]. ASV tables generated from these files are available in /OriginalFiles at https://osf.io/6ku49/. Input files for the analyses presented here are available in /ModifiedFiles at the same link. Scripts necessary to reproduce these analyses are available at https://github.com/fletchec99/UCYNA_at_SPOT.
