# Supplementary material for: Symbiotic UCYN-A strains co-occurred with El Niño, relaxed upwelling, and varied eukaryotes over 10 years off Southern California
Source: ISME Commun. 2023 Jun 24;3:63. doi: 10.1038/s43705-023-00268-y (PMC10290647; doi:10.1038/s43705-023-00268-y)
Supplement: Supplementary file 1 — Supplementary Information [file 43705_2023_268_MOESM1_ESM.pdf]

Symbiotic diazotrophic UCYN-A strains co-occurred with El Niño, relaxed upwelling, and varied eukaryotes over 10 years off Southern California Bight

Fletcher-Hoppe, C., Yeh, Y.C., Raut, Y., Weissman, J.L., and Fuhrman, J.

Supplementary Information

Materials and Methods

DNA SEQUENCING AND PROCESSING: 15-20L of seawater was filtered sequentially through an 80μm mesh (removing mesoplankton) a 1μm glass AE filter (Pall, Port Washington, NY) (collecting the larger 1-80μm size fraction), and finally a 0.2 μm Durapore filter (ED Millipore, Billerica, MA) (collecting the 0.2-1 μm smaller size fraction). DNA was extracted from Durapore filters using the phenol-chloroform method described by Fuhrman et al. [72]. DNA was extracted from AE filters via bead beating, followed by a phenol-chloroform protocol, as described in Lie et al. [73]. The V4-V5 hypervariable region of the 16S and 18S rRNA gene was amplified from these extracts using the universal primers (515Y/926R) as reported by Parada et al. [30], which amplify sequences from both eukaryotic and prokaryotic ribosomal RNA genes [31, 33]. Samples were sequenced on either a HiSeq 2500 in PE250 mode or MiSeq PE300 platform at the USC and UC Davis Genome Core facilities at a target depth of 100,000 sequences/ sample. Per-sample sequence files were submitted to the EMBL database under accession number PRJEB48162 and PRJEB35673. Sequences were processed into Amplicon Sequence Variants (ASVs), which differ by as little as a single base pair, using Divisive Amplicon Denoising Algorithm v2 (DADA2) implemented in Quantitative Insights Into Microbial Ecology v2 (QIIME2) [74] with scripts available at github.com/jcmcnch/eASV-pipeline-for-515Y-926R. [31]. Prokaryotic and eukaryotic ASVs were taxonomically classified using SILVA 132 in May and July of 2020, respectively.

PRINCIPLE COMPONENT ANALYSES: Principle component analysis (PCA) was used to visualize differences in environmental parameters on dates UCYN-A ASVs were absent (<0.01% of the 16S community) vs. present in relative abundances higher or lower than average. PCA was conducted on abiotic data, which was centered and scaled, using prcomp() in R. Ordinations were plotted using autoplot() from the "ggfortify" package in conjunction with ggplot2 [41].

MODELING EFFECTS OF ENVIRONMENTAL PARAMETERS: Sparse binomial regression was used to resolve which environmental parameters best predicted whether UCYN-A1 and UCYN-A2 would be present at SPOT. Model input data consisted of bacteria production rates, nutrient availability, upwelling indices, and other environmental variables. Data from missing dates were linearly interpolated via na.approx() from the R package zoo [42]. UCYN-A ASVs were considered "present" on dates that they were over 0.01% of the 16S community, and "absent" when their relative abundances were lower than 0.01%. For each ASV, a sparse binomial logistic regression model was constructed via the glmnet package in R [75, 76]. 80% of the data was used as training data for the model, and 20% was used as the test set. Variable selection was performed using lasso regression, and the appropriate lambda was selected using 10-fold cross validation on the training set. F1, sensitivity, specificity, and accuracy of each model were calculated on the test set using the caret package [76].

DATA NORMALIZATION: Our DADA2 pipeline splits 16S and 18S sequences, generating separate tables of ASVs for prokaryotes and eukaryotes. In order to plot UCYN-A and associated eukaryotes with the same denominator, sequencing data was normalized as follows. Sequences

from chloroplasts and metazoans were removed, leaving only SSU sequences from prokaryotes and single-celled eukaryotes in the dataset. Raw sequencing counts of prokaryotic and eukaryotic ASVs were divided by the percent of sequences passing quality control in DADA2. Because HiSeq and MiSeq platforms have been shown to discriminate against the 18S rRNA sequences, favoring the shorter 16S rRNA sequences with a two-fold bias [77], sequence counts from eukaryotic ASVs were then doubled. Normalized sequencing counts of prokaryotic and eukaryotic ASVs were combined and converted to proportions, representing the relative abundances of taxa out of the entire microbial community (16S+18S sequences). This method was developed and successfully tested on mixed mock communities, which contain 16S and 18S rRNA sequences in equal concentrations [77], that were sequenced via HiSeq or MiSeq. Following normalization, communities contained equal proportions of each of the organisms in the sequenced sample, as expected (Figure S11). Code normalizing the 16S/ 18S ASV tables of this QIIME-2 pipeline [31] is available at https://github.com/fletchec99/normalizing_16S_18S_tags.

Results and Discussion

PRINCIPLE COMPONENT ANALYSES: Principle component analyses (PCA) indicate that temperature drove variation in the abiotic factors along PC1, which was generally associated with UCYN-A1 and host presence. Higher MEI (Multivariate ENSO Index) was also associated with UCYN-A1 and host presence in PCA. Upwelling indices, as well as indirect indicators of upwelling such as increased nutrient concentration, bacterial production, and chlorophyll concentration, drove variation along PC2 and were associated with UCYN-A1 and host absence. These trends were not as obvious for UCYN-A2 (Figure S3).

MODELING EFFECTS OF ENVIRONMENTAL PARAMETERS: Sparse binomial logistic regression indicated that UCYN-A1 presence was negatively affected by upwelling and no other variables (coefficient= -0.570, sensitivity=0.667, specificity=0.333, accuracy=0.542, F1=0.645). Models were not able to reliably predict the presence of UCYN-A2 (sensitivity=1.00, specificity=0.00, accuracy= 0.5, F1=0.667).

RELATIONSHIP WITH INORGANIC NUTRIENTS: UCYN-A ASVs were not significantly correlated with inorganic nitrogen and phosphorus concentrations (Table S1, Figure 4). Others have observed UCYN-A abundances and activity have no strong relationships with nitrogen concentrations (e.g. [18, 70, 78]). Due to the well-established link between upwelling and increased inorganic nutrient concentrations (e.g. [35]), the strong influence of upwelling on UCYN-A1 might seem incongruous with the weak influence of inorganic nutrients. It is important to note that upwelling indices are aggregated across latitude on a monthly basis, whereas the inorganic nutrients were measured at SPOT on the day of sampling. Upwelling in Southern California is generally coastal, and it is likely that coastal phytoplankton close to the sites of upwelling consumed the upwelled nitrogen and phosphate, before these compounds could reach our study site, ~16km from the coast (Figure 1).

Figures

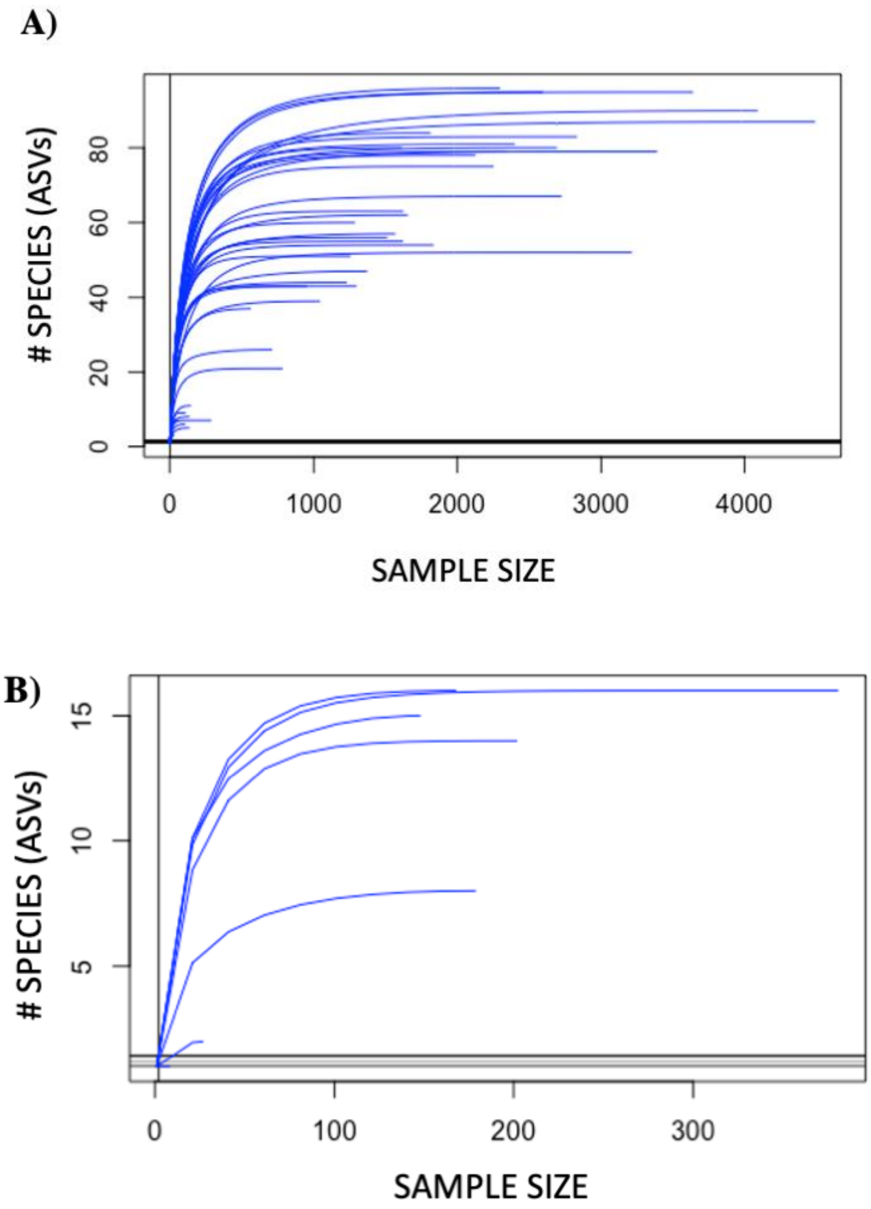

Supplementary Figure 1–Rarefaction curves of AE samples with <100 unique 18S ASVs (species) from the SPOT surface (A) and DCM (B). Five AE samples from the surface and two AE samples from the DCM were excluded from analyses due to insufficient diversity of 18S sequences. All samples with >100 unique 18S ASVs sufficiently captured the diversity of 18S organisms. Rarefaction curves were generated with the R package vegan version 2.5-6.

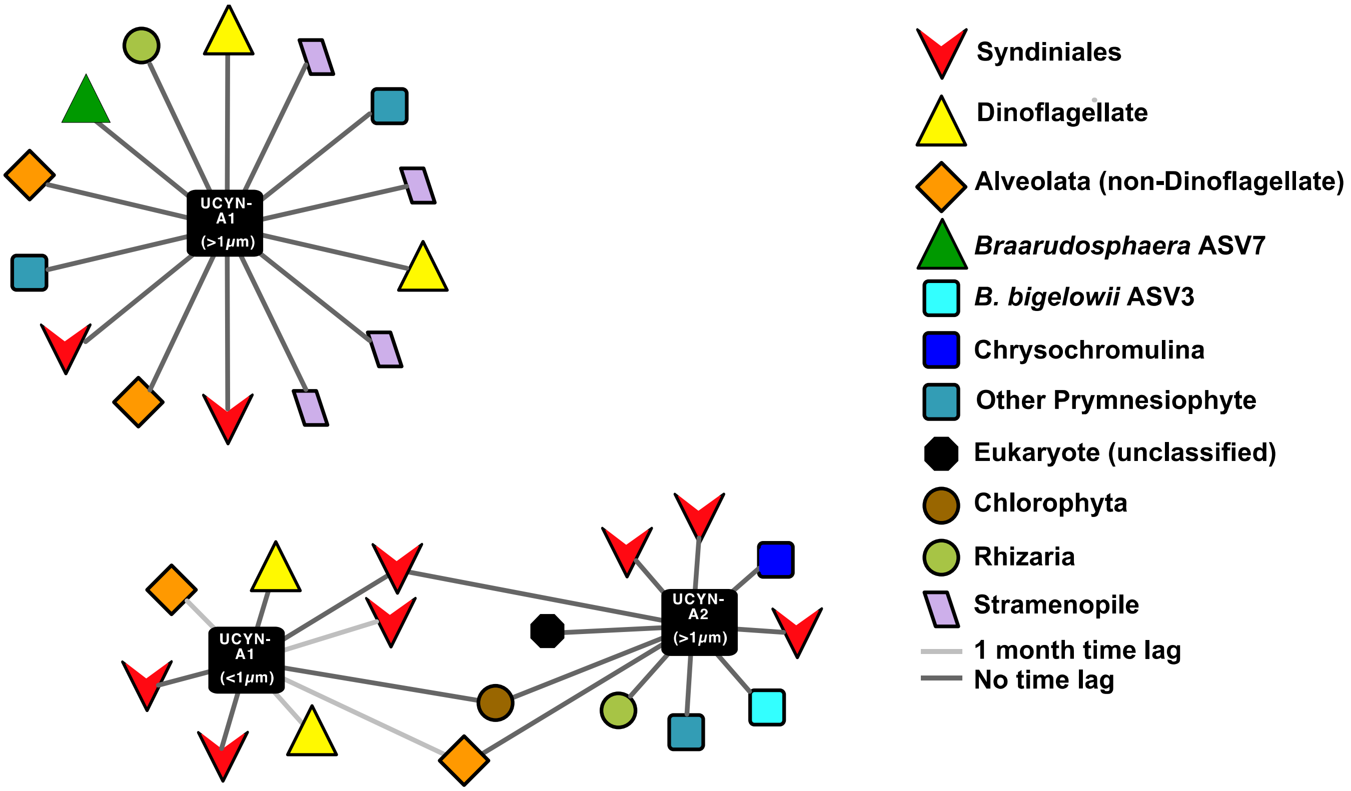

Supplementary Figure 2–eLSA networks constructed using interpolated, non-CLR transformed data from the SPOT surface (5m depth) miss interactions between UCYN-A and other taxa (compare to Figure 5). Networks were generated via eLSA and visualized in Cytoscape 3.5.

Supplementary Table 1–Identifiers for UCYN-A and Braarudospharea ASVs analyzed in this study. Hashes were generated by QIIME2 and were named in alphabetical order (e.g. UCYN-A ASV1 is first alphabetically in the list of six UCYN-A ASVs); representative sequences were classified using SILVA132. BLAST identity is given for the sequences that 100% match an established reference sequence for UCYN-A or its host. Full 16S and 18S sequences are publicly available (see Data Availability Statement).

**QIIME2 generated hash**	**ASV name**	**BLAST Identity (if applicable)**
3d852410f44d21c92c9c55fbbb25187e	UCYN-A SPOT ASV1 or UCYN-A1	100% identical to established UCYN-A1 16S sequence
42c9bf8576acc275f3c9281e6b24f5a3	UCYN-A SPOT ASV2	
6115eab19c52bc45c6ba11d72ec88031	UCYN-A SPOT ASV3	
a641110da9fb0da8f68143b5a79ba5d1	UCYN-A SPOT ASV4	
af1bb1f9fb1c3f3d18571e711df407bb	UCYN-A SPOT ASV5 or UCYN-A2	100% identical to established UCYN-A2 16S sequence
e6f42c535cf3849e1f1e12e7575561b7	UCYN-A SPOT ASV6	
04926e2fd1b8706b4866c02650f702dd	*Braarudospharea bigelowii* SPOT ASV1	100% identical to established UCYN-A1 host 18S sequence
529269deeb5fb7fbf0d0ebda989d9d82	*Braarudospharea bigelowii* SPOT ASV2	
70a5283da28db501a349c5beb22881e7	*Braarudospharea bigelowii* SPOT ASV3	
8c144683114fbb1ad2e9425f7dcd1b02	*Braarudospharea bigelowii* SPOT ASV4	
324627f7f367298bbb5692fc5038e680	*Braarudosphaera* SPOT ASV5x	
ab5338a49f7e9307027c50b3256a7f59	*Braarudospharea* SPOT ASV6x	
be3cdecefbceb0d8b25a2e42ed058b50	*Braarudospharea* SPOT ASV7x	

Supplementary Table 2–Differences in environmental factors on dates UCYN-A1, UCYN-A2, hosts, and potential predator *Lepidodinium* ASVs are present vs. absent. Ekman transport is measured on the Cartesian coordinate system, such that movement Northwards and Eastwards is recorded as a more positive value. Positive MEI indicates El Niño events, while negative MEI represents La Nina conditions. P values were corrected for multiple testing via Benjamini-Hochberg correction. Boldface text indicates $p<0.05$, boldface, underlined text indicates $p<0.01$.

		UCYN-A1 (ASV1)			***Braarudosphaera* ASV7x**			**UCYN-A2 (ASV5)**			***B. bigelowii* ASV3**				***Lepidodinium* ASV**		
		Mean	Standard Error	P-values	Mean	Standard Error	P-values	Mean	Standard Error	P-values	Mean	Standard Error	P-values		Mean	Standard Error	P-values
[NO2+NO3] (uM)	Present	0.471	0.288	0.809	0.514	0.327	0.957	0.626	0.333	0.988	0.603	0.217	0.746	Present	0.620	0.355	0.754
	Absent	0.562	0.164		0.531	0.152		0.462	0.135		0.494	0.260		Absent	0.477	0.144	
[PO4] (uM)	Present	0.253	0.042	0.282	0.247	0.045	0.417	0.222	0.023	0.988	0.179	0.041	0.197	Present	0.246	0.048	0.754
	Absent	0.200	0.014		0.208	0.017		0.221	0.028		0.239	0.011		Absent	0.209	0.016	
Bacterial Production (cells/mL/day)	Present	284052.200	27744.452	**0.006**	305718.400	30237.819	**0.050**	365066.200	44642.812	0.988	329817.400	63965.889	0.197	Present	298758.800	23202.408	0.106
	Absent	481256.900	46408.102		453865.400	44129.048		422600.000	41673.916		428844.800	22388.304		Absent	451901.200	43959.033	
MODIS [Chl] (mg * m-3)	Present	0.507	0.070	0.350	0.451	0.046	0.124	0.509	0.051	0.988	0.444	0.377	0.197	Present	0.566	0.080	0.754
	Absent	0.901	0.286		0.902	0.266		0.883	0.277		0.858	0.030		Absent	0.827	0.255	
MODIS SST (ºC)	Present	19.090	0.400	**0.004**	19.079	0.431	**0.025**	18.878	0.347	0.446	19.242	0.415	0.059	Present	19.197	0.451	**0.029**
	Absent	17.604	0.247		17.724	0.247		17.796	0.292		17.799	0.260		Absent	17.714	0.241	
CUTI index	Present	0.326	0.019	**0.004**	0.345	0.021	**0.025**	0.416	0.026	0.988	0.360	0.033	0.197	Present	0.397	0.026	0.802
	Absent	0.458	0.023		0.437	0.023		0.396	0.022		0.421	0.016		Absent	0.407	0.022	
BEUTI index	Present	0.378	0.098	**0.004**	0.368	0.102	**0.025**	0.680	0.159	0.988	0.321	0.186	0.059	Present	0.630	0.163	0.719
	Absent	1.021	0.130		0.977	0.124		0.806	0.110		0.933	0.057		Absent	0.821	0.110	
MEI	Present	-0.024	0.133	**0.025**	-0.027	0.152	**0.049**	-0.204	0.145	0.988	-0.034	0.162	0.197	Present	-0.183	0.160	0.754
	Absent	-0.467	0.118		-0.431	0.110		-0.336	0.116		-0.386	0.114		Absent	-0.337	0.110	
Magnitude of Surface Wind (m/s)	Present	6.850	0.255	**0.004**	6.881	0.253	**0.025**	7.468	0.314	0.988	7.057	0.354	0.197	Present	7.284	0.297	0.718
	Absent	8.094	0.244		7.980	0.243		7.657	0.231		7.796	0.199		Absent	7.736	0.235	
East-West Component of Ekman Transport (kg/m/s)	Present	-675.851	61.569	**0.004**	-712.083	58.921	**0.025**	-897.779	83.521	0.988	-774.193	100.728	0.197	Present	-815.777	70.719	0.719
	Absent	-1039.232	71.392		-990.841	71.243		-885.622	65.968		-937.017	52.125		Absent	-928.030	68.823	
North-South Component of Ekman Transport (kg/m/s)	Present	-512.139	84.007	**0.004**	-536.981	80.845	**0.025**	-767.567	101.137	0.988	-583.699	119.591	0.197	Present	-708.573	92.885	0.754
	Absent	-919.728	82.514		-874.361	83.054		-743.356	79.260		-820.665	65.127		Absent	-775.010	81.202	

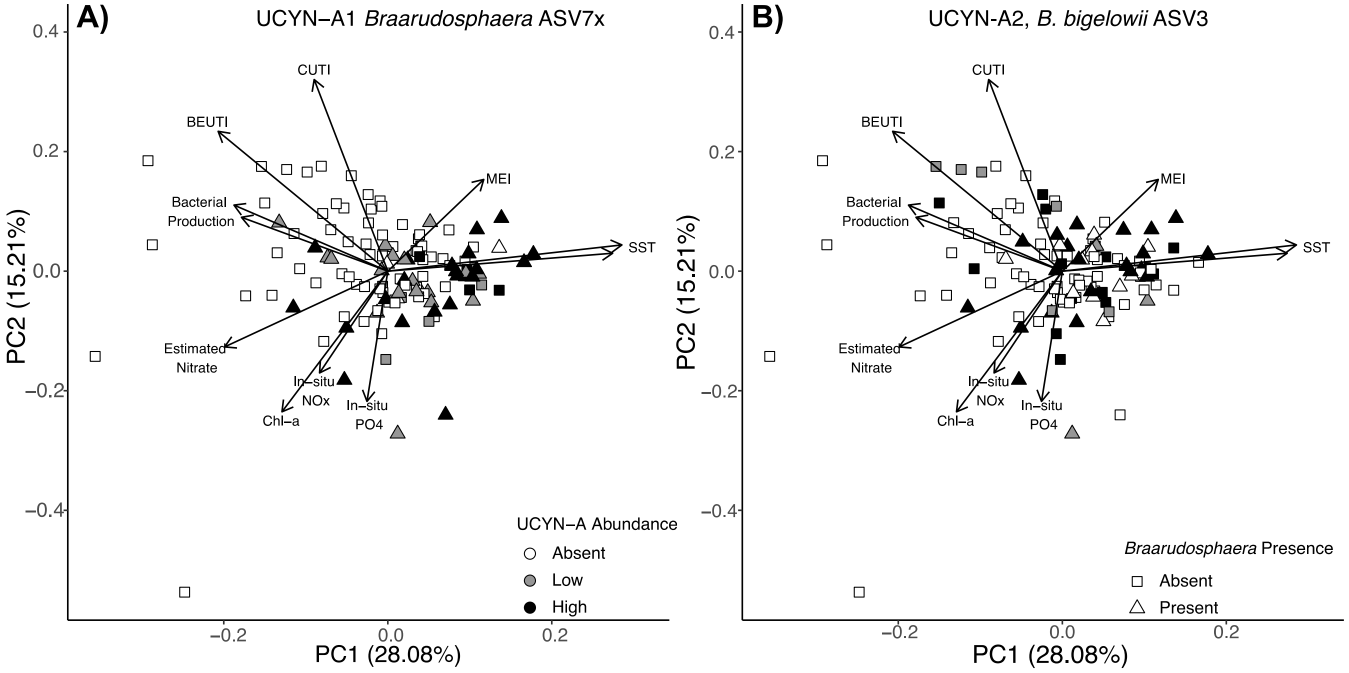

Supplementary Figure 3—Principal component analysis of environmental variables at the SPOT surface, overlaid with UCYN-A1 relative abundance and host presence/ absence (A) and UCYN-A2 relative abundance and host presence/ absence (B), show that temperature and MEI associate strongly with high relative abundance of UCYN-A1, but less strongly with that of UCYN-A2. UCYN-A ASVs were considered "absent" if they were <0.01% of the 16S community, "low abundance" if they were present in abundances lower than average (0.099% for ASV1, UCYN-A1, and 0.026% for ASV5, UCYN-A2), and "high abundance" if their relative abundance was greater than average. This is indicated by white, grey, and black points, respectively; host absence/ presence is indicated by squares vs. circles on both panels.

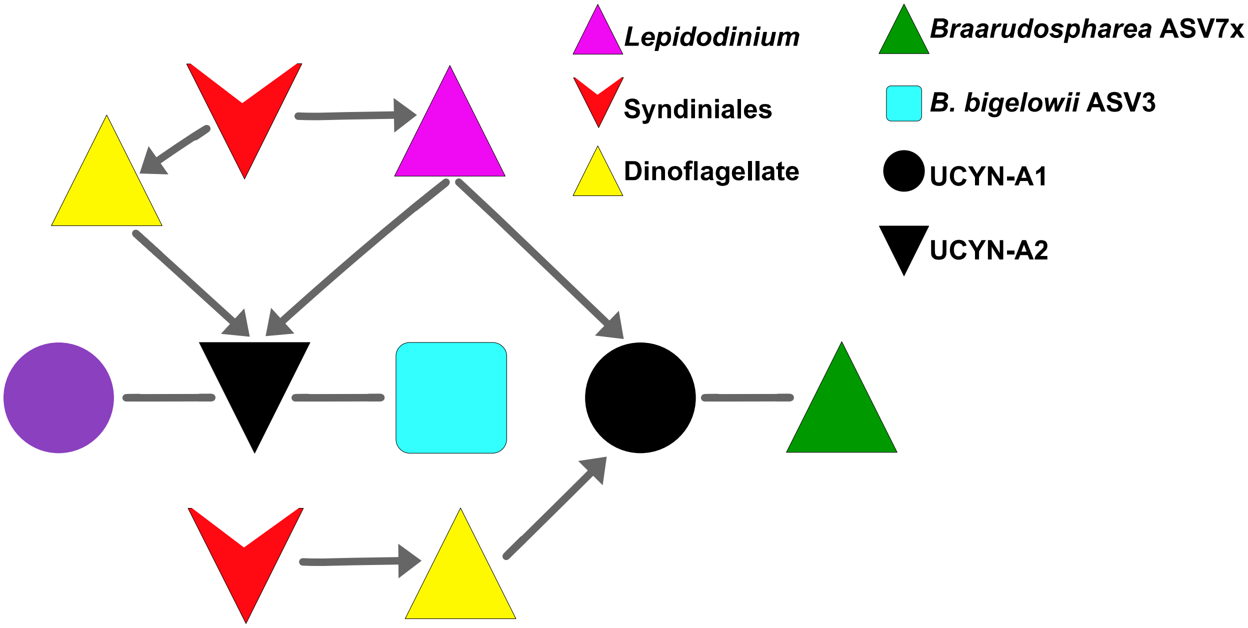

Supplementary Figure 4–Schematic of a hypothetical food web between UCYN-A ASVs and the 18S taxa with which they co-occur at the SPOT surface (see Figure 5). Arrows indicate predation/ parasitism; lines indicate symbiosis.

Supplementary Table 3–UCYN-A ASVs co-occur with a variety of 18S taxa. Hashes were generated via QIIME2; representative sequences were classified using SILVA132. Full 16S and 18S sequences are publicly available (see Data Availability Statement). Also see Supplementary Table 2 for hashes associated with UCYN-A and *Braarudosphaera* ASVs.

Taxonomy	ASV Hash	Co-occurs with
Alveolata	636f2ad7bbc9624ed6d8bd438d96f7b7	UCYN-A1_0.22-1um
Alveolata	b930d2802540ff419424186646952b7d	UCYN-A1_0.22-1um
Alveolata	3d3aaf7802f4db6f8bc887bb9e7774a8	UCYN-A1_1-80um
Alveolata	4b7935f69f43802ce9ea4b65a741845d	UCYN-A1_1-80um
Alveolata	8e0bc96f64c6b0ca6e4aead874edee2a	UCYN-A1_1-80um
Alveolata	b6d8ad4a0a89749e0427b20d028f317d	UCYN-A1_1-80um
Alveolata	cccd609465491f29a68b22cdb1506556	UCYN-A1_1-80um
Alveolata	8a5ef38b1ab34ac0febbd7832a3ed0d1	UCYN-A1_1-80um, UCYN-A1_0.22-1um
Alveolata	2a77e539d5c3d4878fb0cc41c84a0fa3	UCYN-A1_1-80um, UCYN-A1_0.22-1um, UCYN-A2
Alveolata	a9666cf5e782f71ad780c73f0f59c119	UCYN-A2
Braarudosphaera_sp_7x	be3cdecefbceb0d8b25a2e42ed058b50	UCYN-A1_1-80um, UCYN-A1_0.22-1um
Braarudospharea_bigelowii_3	70a5283da28db501a349c5beb22881e7	UCYN-A1_1-80um, UCYN-A2
Chlorophyta	fd7bca02dc31e29cb36624e39cbcc27a	UCYN-A1_1-80um, UCYN-A1_0.22-1um
Chlorophyta	c3b3a5a8e14ea2c9cdb58028bc55bca0	UCYN-A1_1-80um, UCYN-A1_0.22-1um, UCYN-A2
Chrysochromulina	d55992e6da65321a9b3c0ce3426e73ac	UCYN-A1_1-80um, UCYN-A2
Chrysochromulina	eaaf40a3c970e0ec2167de48c4b001eb	UCYN-A2
Dinoflagellate	26cc4edeb2a1788fc8fdf147923c06b4	UCYN-A1_0.22-1um
Dinoflagellate	997d8cc0ee56b8f185884eb47f7bbbd6	UCYN-A1_0.22-1um
Dinoflagellate	4bb7bd73a0c0f6a55e0c4acecdbfd099	UCYN-A1_1-80um
Dinoflagellate	70241d94140d1eaea54e19bc52b1bdd1	UCYN-A1_1-80um
Dinoflagellate	791b0b5d59992db5031a18510b325ced	UCYN-A1_1-80um
Dinoflagellate	ead0d51affbef7dd17d17e483eb0f244	UCYN-A1_1-80um
Dinoflagellate	ef4dcdee685b3fd3427dff66b3a9e379	UCYN-A1_1-80um
Dinoflagellate	f383ee229e9cae5c76c76062e8a0f99f	UCYN-A1_1-80um
Dinoflagellate	a2bcca80cacedb46e9e55cd424022684	UCYN-A1_1-80um, UCYN-A2
Dinoflagellate	dc228f26735fb8483e2e7a8f07994c23	UCYN-A1_1-80um, UCYN-A2
Dinoflagellate	23f2bbf2ecbae94d2b57c207aa8abc62	UCYN-A2
Dinoflagellate	95e7881e06d651005303f80a0824f7f9	UCYN-A2
Dinoflagellate	eaee3f386d9d2d2c4ea3d90ccf81f48a	UCYN-A2
Dinoflagellate	ec296e5b3180d5c65f9e81e7165a4763	UCYN-A2
Eukaryote	0edea9a57e97085c27800904bc55437d	UCYN-A1_1-80um
Eukaryote	af7721ace95e845243e2b715dbcca683	UCYN-A1_1-80um
Eukaryote	72ad5bae980d6f3a205494139435e4e5	UCYN-A1_1-80um, UCYN-A1_0.22-1um
Lepidodinium	c114523e0bef5840b096693e46f441a2	UCYN-A1_1-80um, UCYN-A1_0.22-1um, UCYN-A2
Prymnesiophyte	23206df8663c546b75a92fadb5de9b33	UCYN-A1_0.22-1um
Prymnesiophyte	79c87d3c4954a1dec1972fc679befcb5	UCYN-A1_1-80um
Prymnesiophyte	98d1369a93242f295deda7ee996b9886	UCYN-A1_1-80um
Prymnesiophyte	803aa95deeb6167672a24a67029f2b82	UCYN-A1_1-80um, UCYN-A1_0.22-1um
Prymnesiophyte	ffa92571884bcbe0193ce4a1e4e843be	UCYN-A1_1-80um, UCYN-A1_0.22-1um
Prymnesiophyte	55bc25d102de6079cc48ba48515e72e2	UCYN-A2
Rhizaria	307e50f0be54fdc6ac0ea8183ff77d0e	UCYN-A1_0.22-1um
Rhizaria	a615f49d7d2fbed5b85069fb94087fa3	UCYN-A1_1-80um
Rhizaria	ded52caa99b40106f449ecc43dbbeee0	UCYN-A1_1-80um
Rhizaria	4f2711ffbb0611ea359dd96680df1c4f	UCYN-A2
Rhizaria	afacfa595bc8d424f2a648990538e0d4	UCYN-A2
Stramenopile	eac29d65650c1a316e01b9fa8a5a9038	UCYN-A1_0.22-1um
Stramenopile	25e0c3351c5b93e80e0f02e6ba23c077	UCYN-A1_1-80um
Stramenopile	3465aaadf57e9cf04c930da3beb3deef	UCYN-A1_1-80um
Stramenopile	5ca13089ea80484bd62871929d00bf95	UCYN-A1_1-80um
Stramenopile	6f39e2bbe0e4c6fa1e2eb082348de7c5	UCYN-A1_1-80um
Stramenopile	8940d219ac059e1421b8910843b4fd82	UCYN-A1_1-80um
Stramenopile	9ec148397bce05c40a8d97a91574a1ef	UCYN-A1_1-80um
Stramenopile	ddaade5c44a6bbd3a74f879c85b5faf3	UCYN-A1_1-80um
Stramenopile	e67e2f1a5016412cf9826b35523352c4	UCYN-A1_1-80um
Stramenopile	e966740cb469db7493a9b384262bce67	UCYN-A1_1-80um
Stramenopile	a94291b249e004b97f399d41c5cc4b82	UCYN-A1_1-80um, UCYN-A1_0.22-1um
Stramenopile	5d86bdd765a6ce520791f28dda5819ed	UCYN-A1_1-80um, UCYN-A2
Stramenopile	3d03bbd7eb3a06f19d2f377b5eb2efb5	UCYN-A2
Stramenopile	e09f07091bd33255025bd0a1cec414ca	UCYN-A2
Syndiniales	00399bcfe64d51dac93b62a40832b514	UCYN-A1_0.22-1um
Syndiniales	10657154c17634d9006a00243a3736b1	UCYN-A1_0.22-1um
Syndiniales	38caa165f588a30638f74c2edb93662c	UCYN-A1_0.22-1um
Syndiniales	69e6b69c7b0addcc5c9c44d08015e45b	UCYN-A1_0.22-1um
Syndiniales	bce9eb5a6281547d9fffcd95d9156913	UCYN-A1_0.22-1um
Syndiniales	1082ec476ee33ebf0800a137e9dd6730	UCYN-A1_1-80um
Syndiniales	12516469ae16dbe47afd1383f1a38d29	UCYN-A1_1-80um
Syndiniales	43d72e28390647a43a479322275ebabc	UCYN-A1_1-80um
Syndiniales	4f0a7afd789e6f14b745c17acad17068	UCYN-A1_1-80um
Syndiniales	6fa9b449505f9b00f18edbedca877d17	UCYN-A1_1-80um
Syndiniales	80d903e0cb7271e69b2ec8bd1f87f45c	UCYN-A1_1-80um
Syndiniales	b22089acf5e02fe3477be0ae8fe10cb5	UCYN-A1_1-80um
Syndiniales	bf238b09c39ad60be96eb93f30ac6915	UCYN-A1_1-80um
Syndiniales	eefc3152825b60051b3f78c504aca2a9	UCYN-A1_1-80um
Syndiniales	f14396a0e7efc97700da47bcb853bfad	UCYN-A1_1-80um
Syndiniales	365026beab40dc3dacd12678ac56cfc2	UCYN-A1_1-80um, UCYN-A1_0.22-1um
Syndiniales	6bbbadb60359057c81abc84e7d0b797a	UCYN-A1_1-80um, UCYN-A1_0.22-1um
Syndiniales	b3a109d089b15d7d926497af27f7fd2c	UCYN-A1_1-80um, UCYN-A1_0.22-1um
Syndiniales	624af6018837349e853bc2e772461e80	UCYN-A1_1-80um, UCYN-A1_0.22-1um, UCYN-A2
Syndiniales	68216c70c4cc7ae340a7f17cb6973f5b	UCYN-A1_1-80um, UCYN-A1_0.22-1um, UCYN-A2
Syndiniales	6a30306b1212b48152ee097506fedad2	UCYN-A2
Syndiniales	6c3205e48cb5c1cc405609e73063607d	UCYN-A2
Syndiniales	6cf6cfba7e7b46309c2fc38b44a28064	UCYN-A2

Supplementary Figure 5–Relative abundances of UCYN-A 16S sequences linearly correlate with relative abundances of host 18S sequences. A) Pairwise comparison of all UCYN-A vs. all *Braarudospharea* ASVs in the SPOT dataset. B) UCYN-A1 (UCYNA_1) and UCYN-A2 (UCYNA_5) relative abundances correlate with one another, hence these symbionts appear to correlate with one another's host organisms. Relative abundances are given out of the whole microbial community (16S + 18S) in the 1-80um size fraction at 5m depth. See Supplementary Table 2 for hashes associated with UCYN-A and *Braarudosphaera* ASVs.

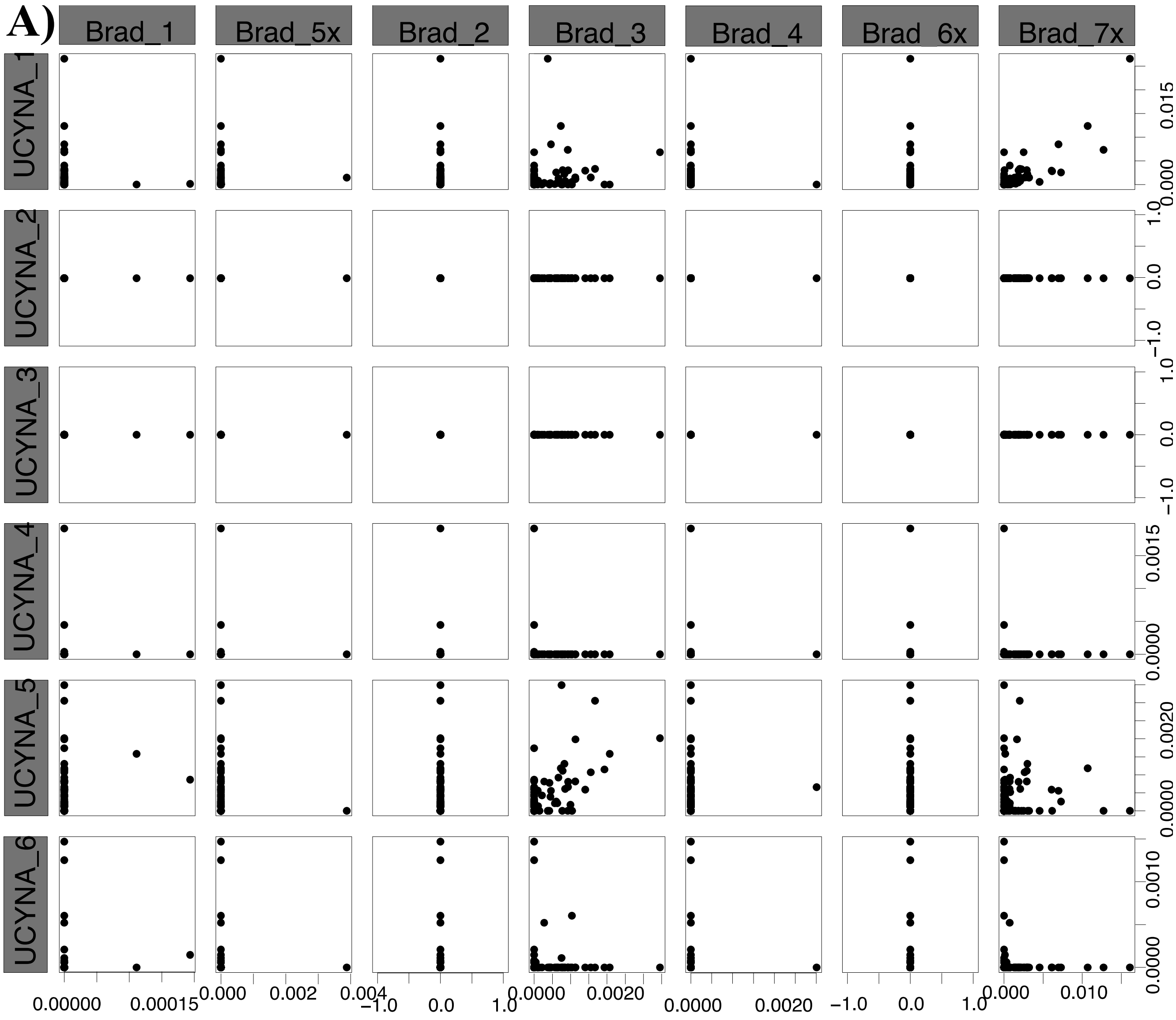

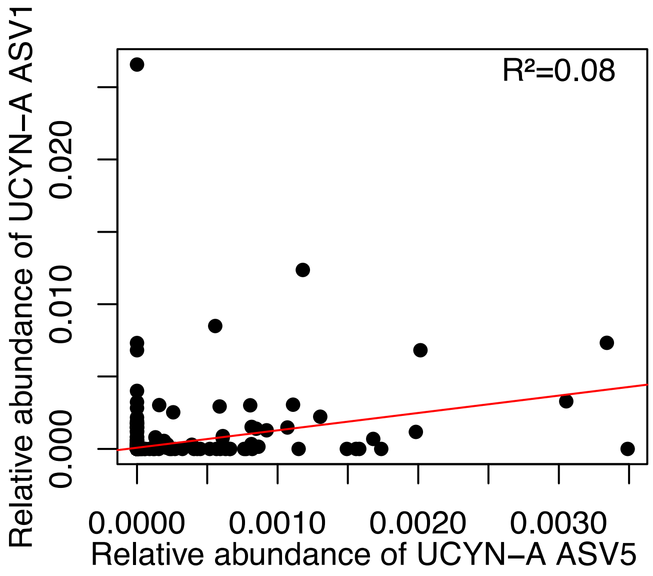
R²=0.08
Relative abundance of UCYN–A ASV1
0.000
0.010
0.020
0.0000
0.0010
0.0020
0.0030
Relative abundance of UCYN–A ASV5

B)

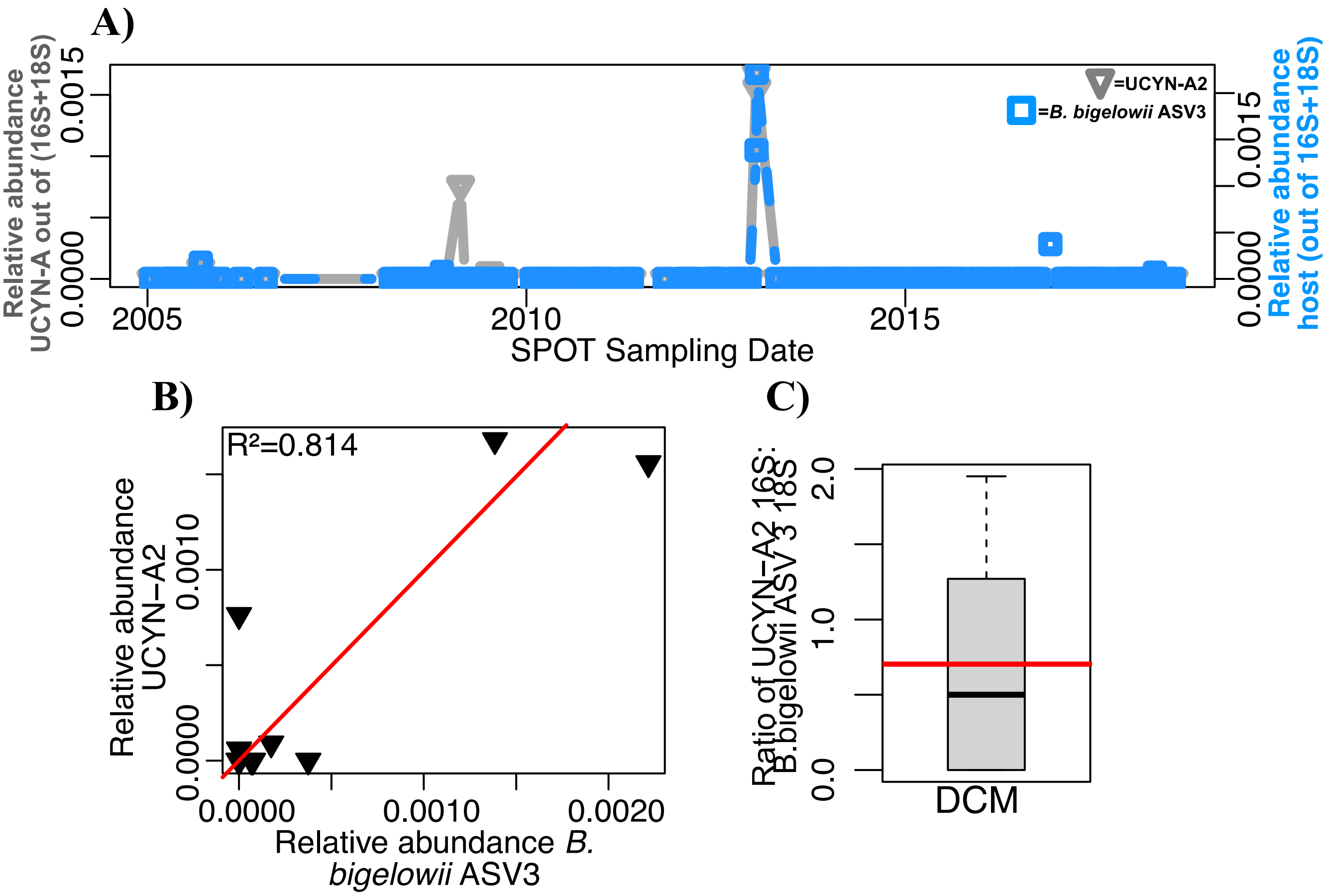
A)
Relative abundance
UCYN-A out of (16S+18S)
0.0000
0.0015
=UCYN-A2
=B. bigelowii ASV3
Relative abundance
host (out of 16S+18S)
2005
2010
2015
SPOT Sampling Date
B)
R²=0.814
Relative abundance
UCYN–A2
0.0000
0.0010
0.0000
0.0010
0.0020
Relative abundance B. bigelowii ASV3
C)
Ratio of UCYN–A2 16S:
B.bigelowii ASV 3 18S
0.0
1.0
2.0
DCM

Supplementary Figure 6–A) UCYN-A2 co-occurs with its most common host at the DCM across the SPOT time series. B) UCYN-A2 relative abundance correlates with its putative host relative abundance at the DCM. C) The ratio of 16S: 18S genes of these organisms is, on average, as expected. Boxplot values indicate the median and IQR values of this ratio; the red line indicates the average (0.703).

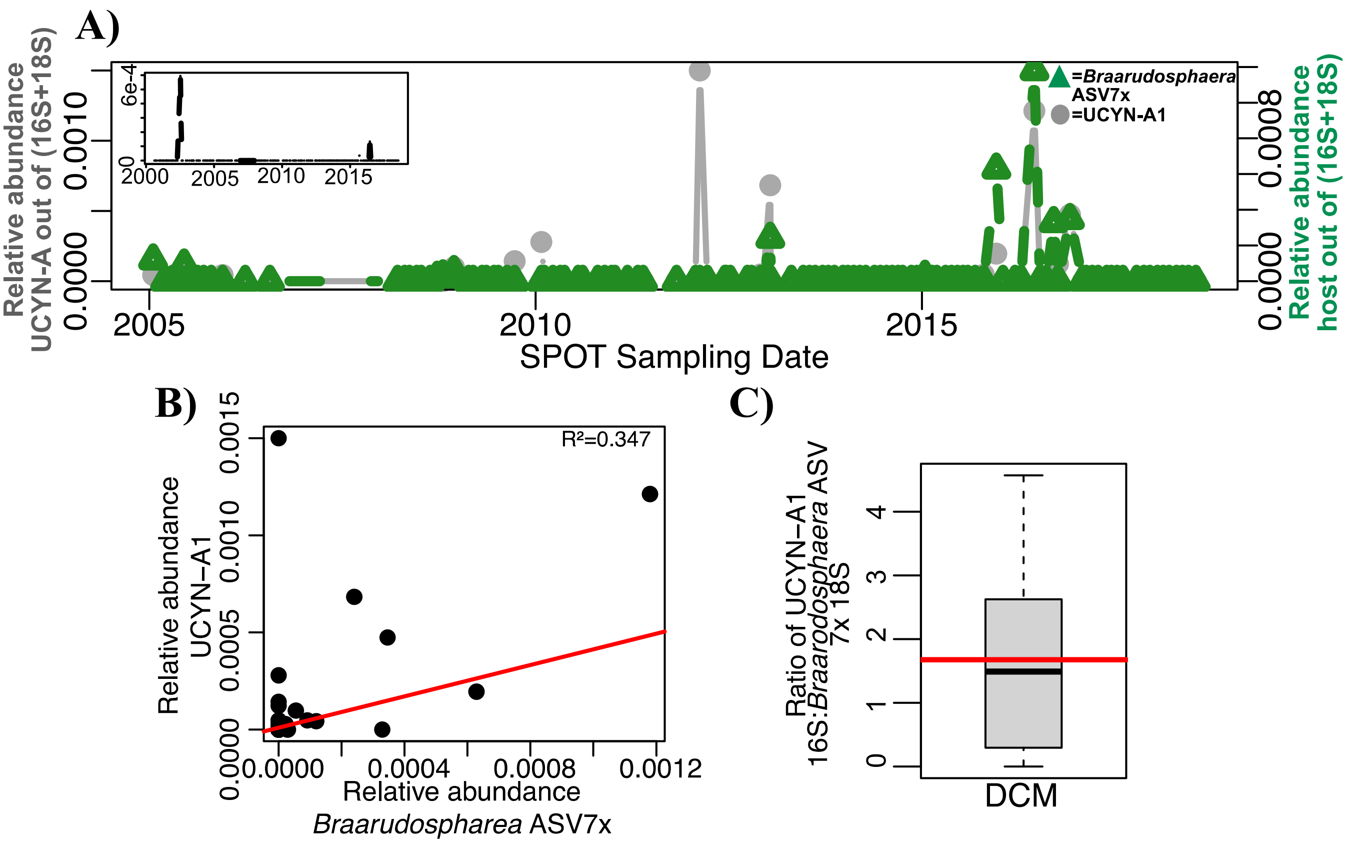

Supplementary Figure 7–A) UCYN-A1 co-occurs with its putative host at the DCM across the SPOT time series. Panel inset indicates the relative abundance of UCYN-A1 in the smaller size fraction. B) UCYN-A1 relative abundance correlates with its putative host relative abundance. C) The ratio of 16S: 18S genes of these organisms is, on average, as expected. Boxplot values indicate the median and IQR values of this ratio; the red line indicates the average (1.674).

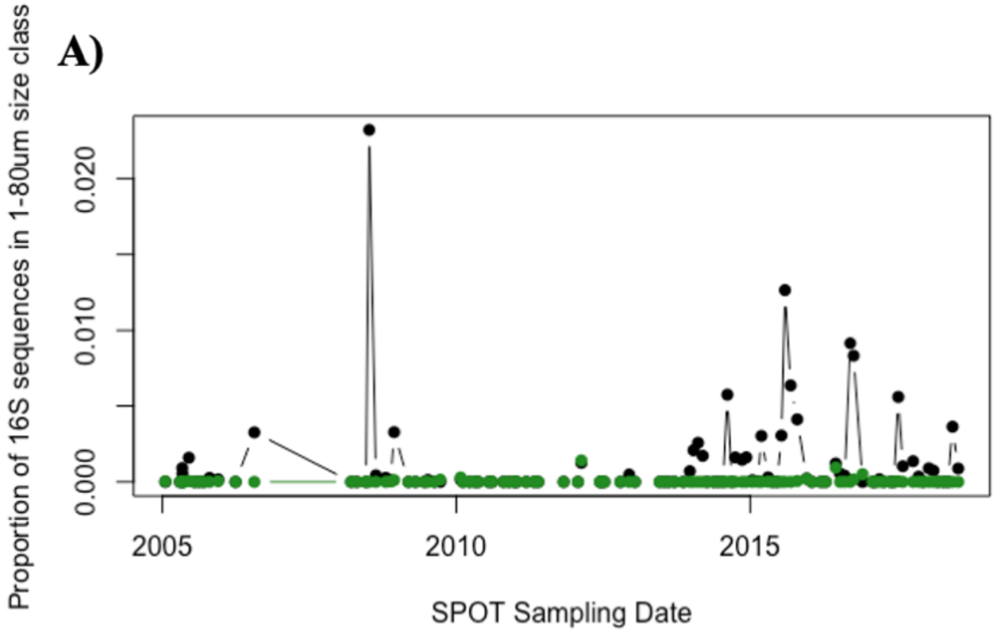
A)
Proportion of 16S sequences in 1-80um size class
0.000
0.010
0.020
2005
2010
2015
SPOT Sampling Date

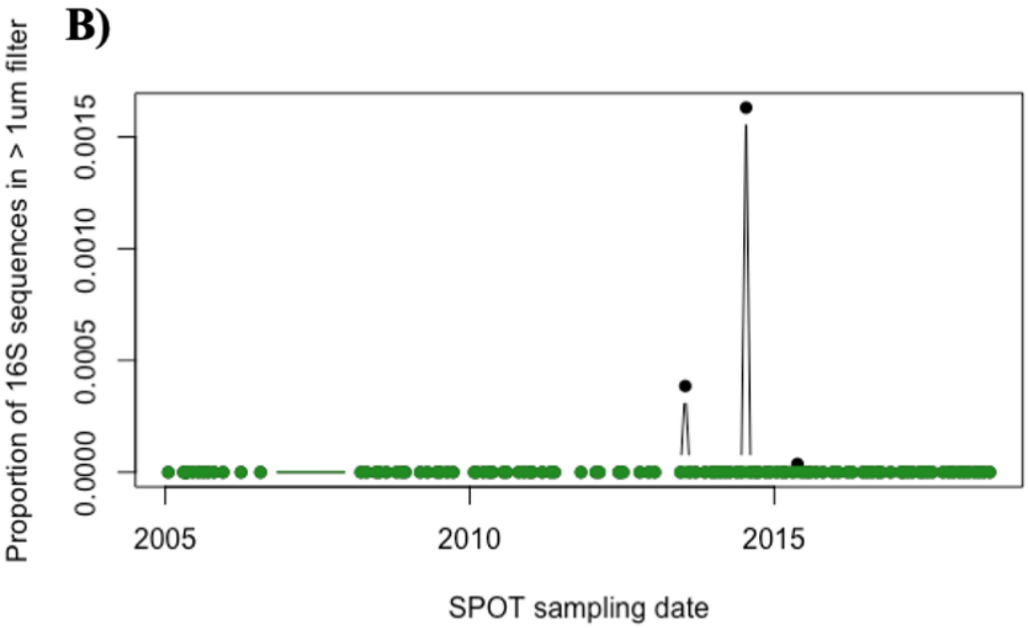
B)
Proportion of 16S sequences in > 1um filter
0.0000
0.0005
0.0010
0.0015
2005
2010
2015
SPOT sampling date

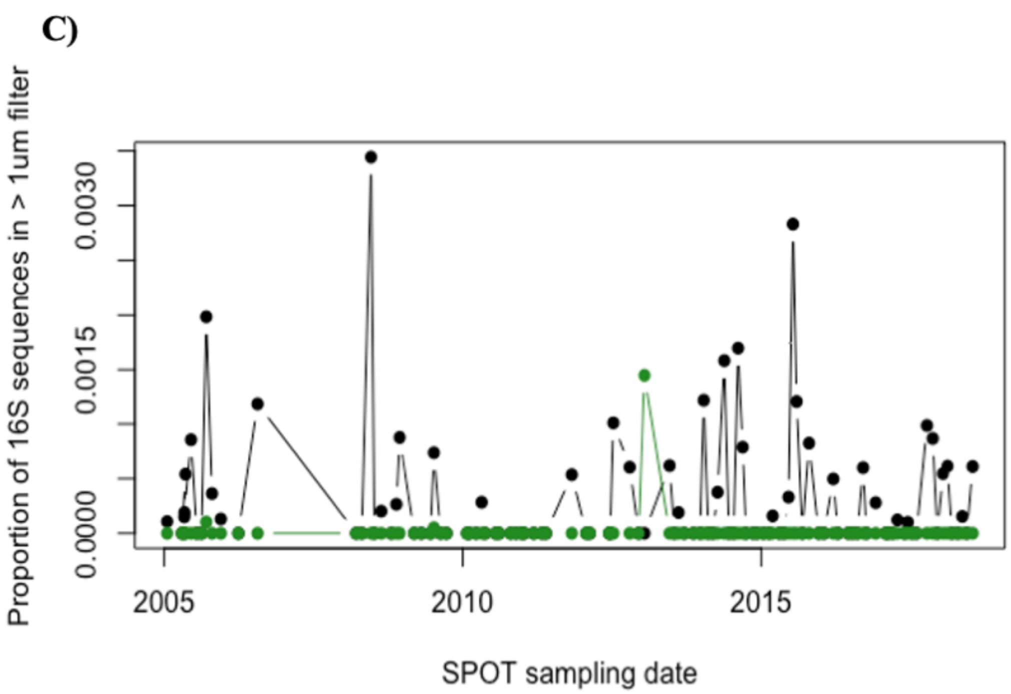

Supplementary Figure 8–Relative abundance of UCYN-A1 (UCYN-A SPOT ASV1) (A), UCYN-A SPOT ASV6 (B), and UCYN-A2 (UCYN-A SPOT ASV5) (C) at the SPOT surface (black) and DCM (green) over time. Relative abundances are given out of the 16S community in the larger size fraction (AE filters; 1-80um).

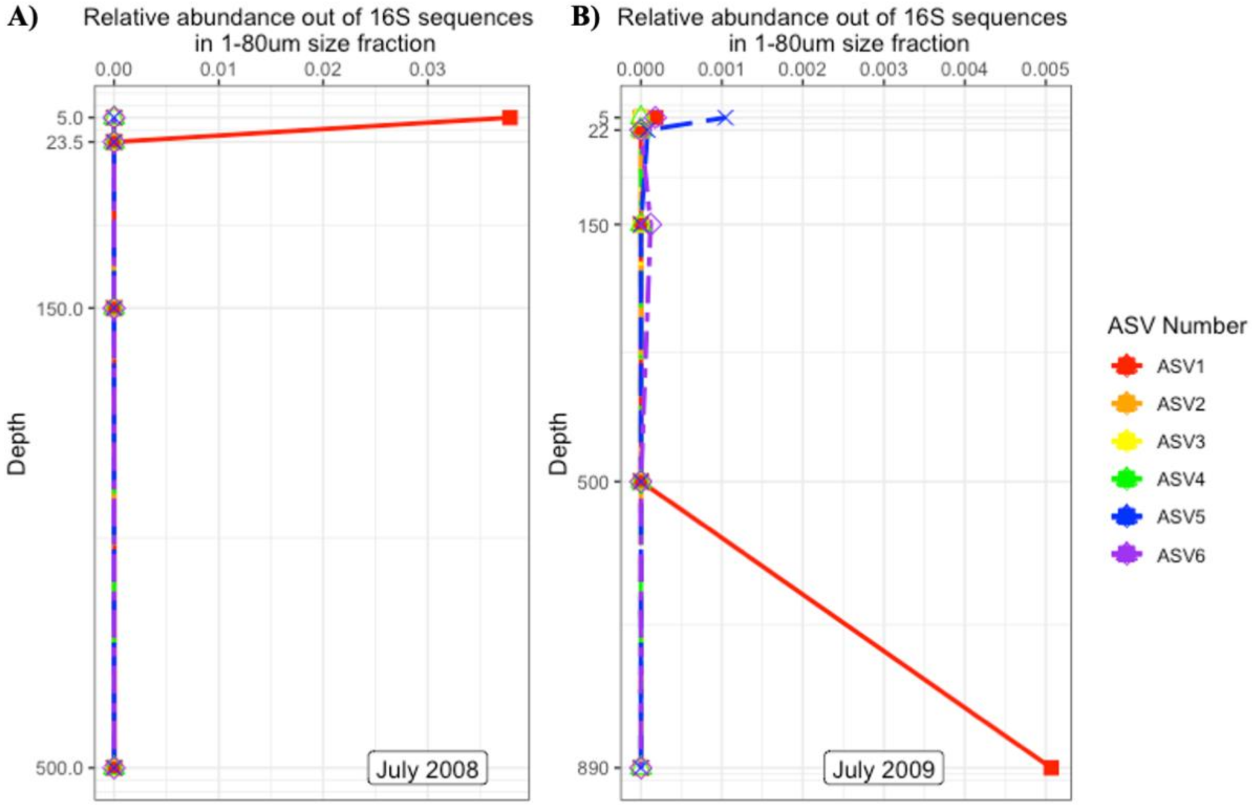

Supplementary Figure 9–Relative abundance of UCYN-A ASVs in the larger size fraction over depth in A) July 2008, the date UCYN-A1 reached its maximum relative abundance, and B) July 2009, the date UCYN-A1 appeared at 890m.

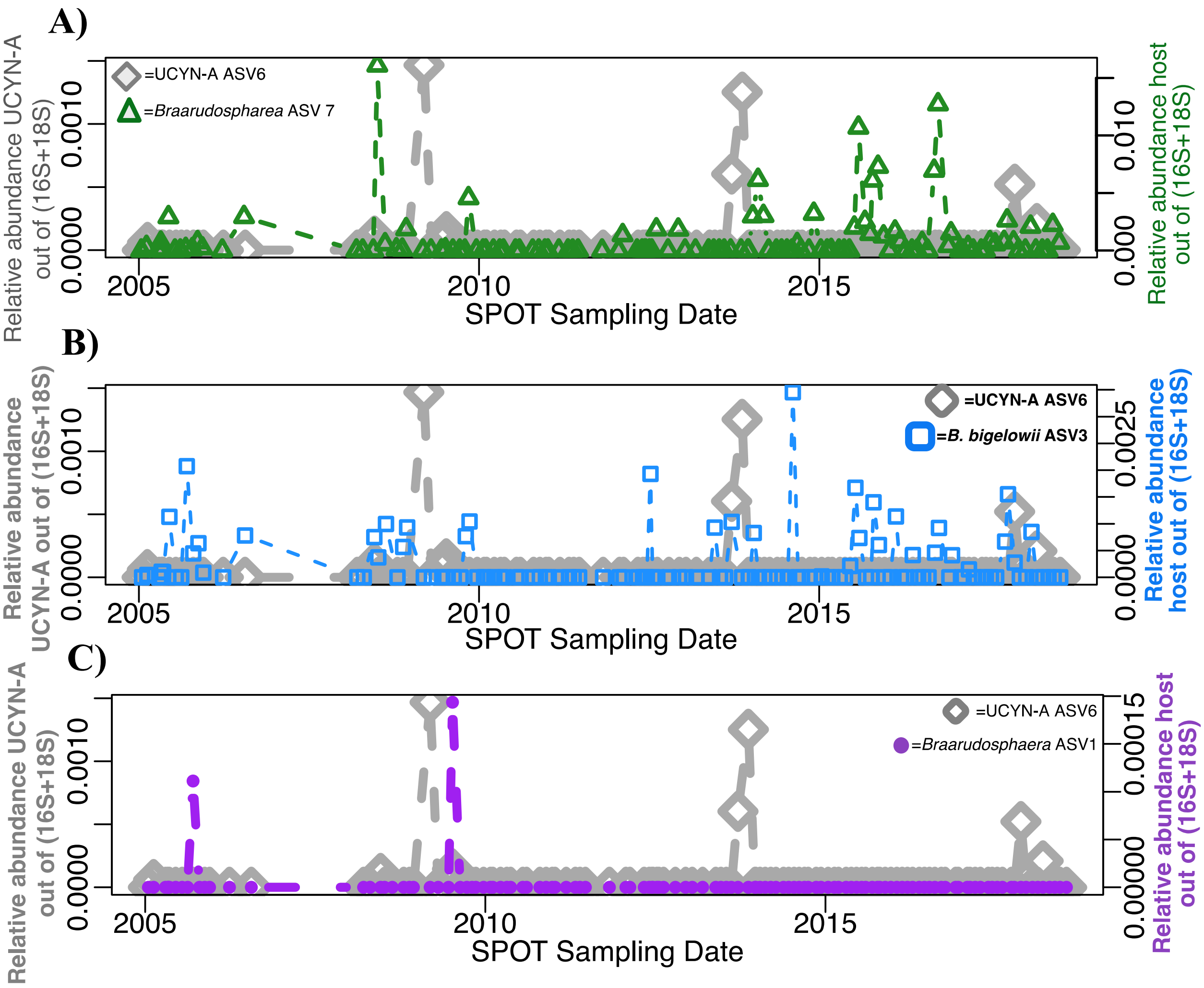

Supplementary Figure 10–UCYN-A ASV6 does not co-occur with any *Braarudosphaera* ASV across the SPOT dataset at 5m. See Supplementary Table 2 for hashes associated with UCYN-A and *Braarudosphaera* ASVs.

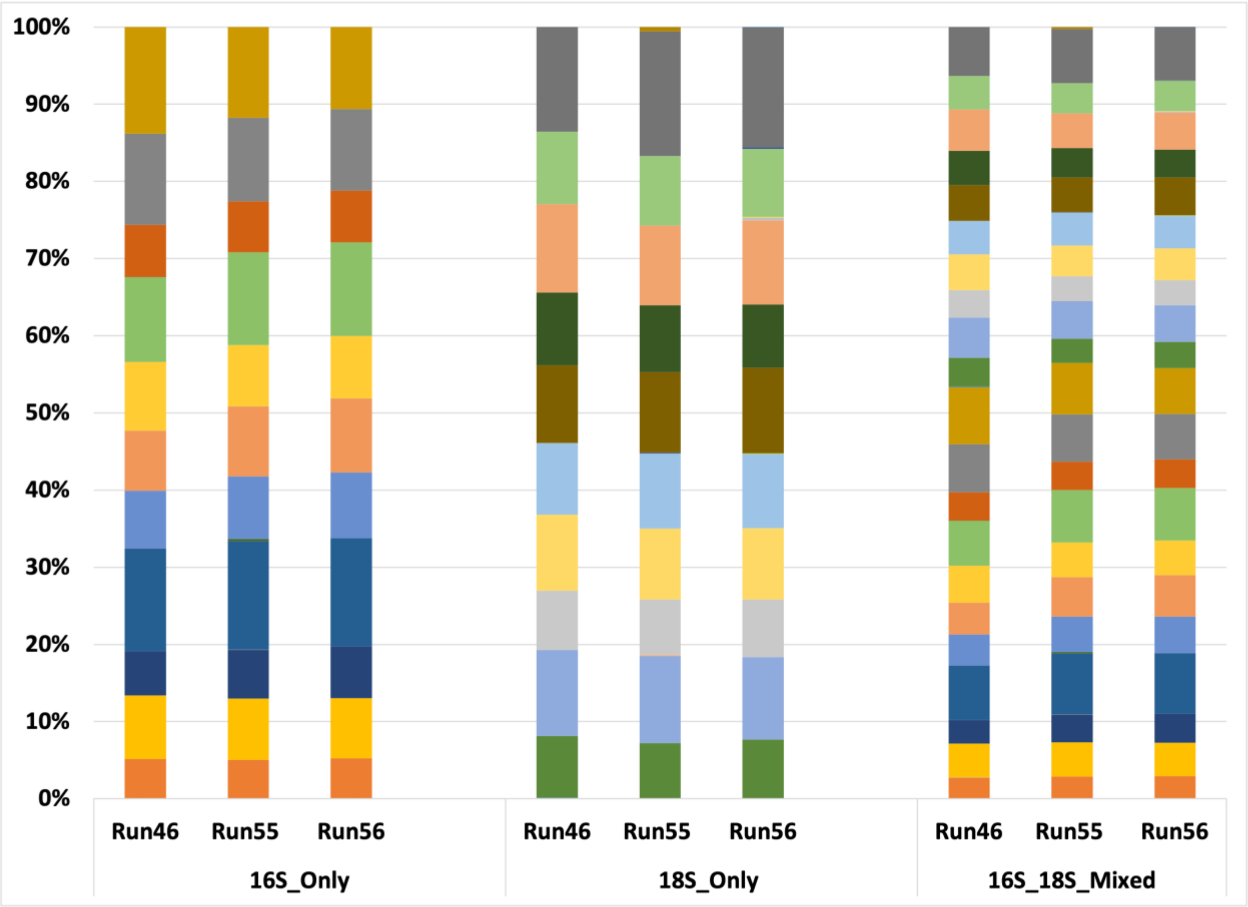

Supplementary Figure 11–Mixed mock communities contain even proportions of 16S (right) and 18S (middle) sequences. DNA from the small subunit of the rRNA gene of 21 organisms was pooled and sequenced in equal concentrations on an Illumina HiSeq or MiSeq. Normalized mixed mock communities match the proportions of sequenced DNA, indicating normalization was successful (left).

<u>Additional References:</u>

72. Fuhrman JA, Comeau DE, Hagstrom A, Chan AM. Extraction from Natural Planktonic Microorganisms of DNA Suitable for Molecular Biological Studies. *Applied and Environmental Microbiology* 1988; **54**: 1426.

73. Lie A, Kim D, Schnetzer A, Caron D. Small-scale temporal and spatial variations in protistan community composition at the San Pedro Ocean Time-series station off the coast of southern California. *Aquatic Microbial Ecology* 2013; **70**.

74. Callahan BJ, McMurdie PJ, Rosen MJ, Han AW, Johnson AJ, Holmes SP. DADA2: High-resolution sample inference from Illumina amplicon data. *Nat Methods* 2016; **13**: 581–583.

75. Friedman J, Hastie T, Tibshirani R. Regularization Paths for Generalized Linear Models via Coordinate Descent. *Journal of Statistical Software* 2010; **33**: 1–22.

76. Simon N, Friedman J, Hastie T, Tibshirani R. Regularization Paths for Cox's Proportional Hazards Model via Coordinate Descent. *Journal of Statistical Software* 2011; **39**: 1–13.

77. Katoh K, Standley DM. MAFFT multiple sequence alignment software version 7: Improvements in performance and usability. *Molecular Biology and Evolution* 2013; **30**: 772–780.

78. Yeh Y-C, Needham DM, Sieradzki ET, Fuhrman JA. Taxon Disappearance from Microbiome Analysis Reinforces the Value of Mock Communities as a Standard in Every Sequencing Run. *mSystems* 2018; **3**.

79. Turk-Kubo KA, Achilles KM, Serros TR, Ochiai M, Montoya JP, Zehr JP. Nitrogenase (nifH) gene expression in diazotrophic cyanobacteria in the Tropical North Atlantic in response to nutrient amendments. *Front Microbiol* 2012; **3**: 386.
